# Epidemiologic Features of Recovery From SARS-CoV-2 Infection

**DOI:** 10.1001/jamanetworkopen.2024.17440

**Published:** 2024-06-17

**Authors:** Elizabeth C. Oelsner, Yifei Sun, Pallavi P. Balte, Norrina B. Allen, Howard Andrews, April Carson, Shelley A. Cole, Josef Coresh, David Couper, Mary Cushman, Martha Daviglus, Ryan T. Demmer, Mitchell S. V. Elkind, Linda C. Gallo, Jose D. Gutierrez, Virginia J. Howard, Carmen R. Isasi, Suzanne E. Judd, Alka M. Kanaya, Namratha R. Kandula, Robert C. Kaplan, Gregory L. Kinney, Anna M. Kucharska-Newton, Daniel T. Lackland, Joyce S. Lee, Barry J. Make, Yuan-I. Min, Joanne M. Murabito, Arnita F. Norwood, Victor E. Ortega, Kelley Pettee Gabriel, Bruce M. Psaty, Elizabeth A. Regan, Daniela Sotres-Alvarez, David Schwartz, James M. Shikany, Bharat Thyagarajan, Russell P. Tracy, Jason G. Umans, Ramachandran S. Vasan, Sally E. Wenzel, Prescott G. Woodruff, Vanessa Xanthakis, Ying Zhang, Wendy S. Post

**Affiliations:** 1 Division of General Medicine, Department of Medicine, Columbia University Irving Medical Center, New York, New York; 2 Department of Biostatistics, Mailman School of Public Health, Columbia University Irving Medical Center, New York, New York; 3 Center for Epidemiology and Population Health, Northwestern Feinberg School of Medicine, Chicago, Illinois; 4 Department of Epidemiology, School of Public Health, University of Alabama at Birmingham; 5 Texas Biomed, San Antonio, Texas; 6 Departments of Medicine and Public Health, NYU Grossman School of Medicine, New York, New York; 7 Collaborative Studies Coordinating Center, Department of Biostatistics, University of North Carolina, Chapel Hill; 8 Division of Hematology/Oncology, Department of Medicine, Larner School of Medicine, University of Vermont, Burlington; 9 Institute for Minority Health Research, University of Illinois College of Medicine, Chicago; 10 Division of Epidemiology, Department of Quantitative Health Sciences, College of Medicine and Science, Mayo Clinic, Rochester, Minnesota; 11 Department of Neurology, Columbia University Irving Medical Center, New York, New York; 12 American Heart Association, Dallas, Texas; 13 Department of Psychology, San Diego State University, California; 14 Department of Epidemiology and Population Health, Albert Einstein College of Medicine, Bronx, New York; 15 Departments of Medicine, Epidemiology, and Biostatistics, University of California, San Francisco; 16 Department of Medicine, Northwestern Feinberg School of Medicine, Chicago, Illinois; 17 Department of Epidemiology, University of Colorado Denver; 18 Department of Epidemiology and Environmental Health, University of Kentucky, Lexington; 19 Department of Neurology, Medical University of South Carolina, Charleston; 20 Division of Pulmonary and Critical Care, Department of Medicine, University of Colorado, Aurora; 21 Division of Pulmonary, Critical Care and Sleep, Department of Medicine, National Jewish Health, Denver, Colorado; 22 Department of Medicine, University of Mississippi Medical Center, Jackson; 23 Department of Medicine, Boston University, Boston, Massachusetts; 24 Department of Medicine, University of Mississippi Medical Center, Jackson; 25 Division of Pulmonary Medicine, Department of Medicine, Mayo Clinic, Phoenix, Arizona; 26 Departments of Epidemiology and Medicine, University of Washington, Seattle; 27 Division of Rheumatology, Department of Medicine, National Jewish Health, Denver, Colorado; 28 Division of Preventive Medicine, Heersink School of Medicine, University of Alabama at Birmingham; 29 Department of Laboratory Medicine and Pathology, University of Minnesota Medical School, Minneapolis; 30 Department of Pathology and Laboratory Medicine, University of Vermont, Burlington; 31 MedStar Health Research Institute, School of Medicine, Georgetown University, Washington, District of Columbia; 32 School of Public Health, University of Texas School of Public Health San Antonio; 33 Department of Pulmonary, Allergy and Critical Care, Department of Medicine, University of Pittsburgh, Pennsylvania; 34 Divison of Pulmonary, Critical Care, Allergy and Sleep Medicine, Department of Medicine, University of California, San Francisco; 35 Section of Preventive Medicine and Epidemiology, Department of Medicine, Boston University School of Medicine, Boston, Massachusetts; 36 Department of Biostatistics, Boston University School of Public Health, Boston, Massachusetts; 37 Departments of Biostatistics and Epidemiology, Hudson College of Public Health, University of Oklahoma Health Sciences Center, Oklahoma City; 38 Division of Cardiology, Departments of Medicine and Epidemiology, Johns Hopkins University, Baltimore, Maryland

## Abstract

**Importance:**

Persistent symptoms and disability following SARS-CoV-2 infection, known as post–COVID-19 condition or “long COVID,” are frequently reported and pose a substantial personal and societal burden.

**Objective:**

To determine time to recovery following SARS-CoV-2 infection and identify factors associated with recovery by 90 days.

**Design, Setting, and Participants:**

For this prospective cohort study, standardized ascertainment of SARS-CoV-2 infection was conducted starting in April 1, 2020, across 14 ongoing National Institutes of Health–funded cohorts that have enrolled and followed participants since 1971. This report includes data collected through February 28, 2023, on adults aged 18 years or older with self-reported SARS-CoV-2 infection.

**Exposure:**

Preinfection health conditions and lifestyle factors assessed before and during the pandemic via prepandemic examinations and pandemic-era questionnaires.

**Main Outcomes and Measures:**

Probability of nonrecovery by 90 days and restricted mean recovery times were estimated using Kaplan-Meier curves, and Cox proportional hazards regression was performed to assess multivariable-adjusted associations with recovery by 90 days.

**Results:**

Of 4708 participants with self-reported SARS-CoV-2 infection (mean [SD] age, 61.3 [13.8] years; 2952 women [62.7%]), an estimated 22.5% (95% CI, 21.2%-23.7%) did not recover by 90 days post infection. Median (IQR) time to recovery was 20 (8-75) days. By 90 days post infection, there were significant differences in restricted mean recovery time according to sociodemographic, clinical, and lifestyle characteristics, particularly by acute infection severity (outpatient vs critical hospitalization, 32.9 days [95% CI, 31.9-33.9 days] vs 57.6 days [95% CI, 51.9-63.3 days]; log-rank *P* < .001). Recovery by 90 days post infection was associated with vaccination prior to infection (hazard ratio [HR], 1.30; 95% CI, 1.11-1.51) and infection during the sixth (Omicron variant) vs first wave (HR, 1.25; 95% CI, 1.06-1.49). These associations were mediated by reduced severity of acute infection (33.4% and 17.6%, respectively). Recovery was unfavorably associated with female sex (HR, 0.85; 95% CI, 0.79-0.92) and prepandemic clinical cardiovascular disease (HR, 0.84; 95% CI, 0.71-0.99). No significant multivariable-adjusted associations were observed for age, educational attainment, smoking history, obesity, diabetes, chronic kidney disease, asthma, chronic obstructive pulmonary disease, or elevated depressive symptoms. Results were similar for reinfections.

**Conclusions and Relevance:**

In this cohort study, more than 1 in 5 adults did not recover within 3 months of SARS-CoV-2 infection. Recovery within 3 months was less likely in women and those with preexisting cardiovascular disease and more likely in those with COVID-19 vaccination or infection during the Omicron variant wave.

## Key Points

**Question:**

What variables are associated with time to recovery from SARS-CoV-2 infection?

**Findings:**

In this cohort study of 4708 participants in a US meta-cohort, the median self-reported time to recovery from SARS-CoV-2 infection was 20 days, and an estimated 22.5% had not recovered by 90 days. Women and adults with suboptimal prepandemic health, particularly clinical cardiovascular disease, had longer times to recovery, whereas vaccination prior to infection and infection during the Omicron variant wave were associated with shorter times to recovery.

**Meaning:**

These findings suggest that interventions to reduce severity of acute infection, such as vaccination, may help to mitigate the increased risk of persistent symptoms observed in women and adults with suboptimal prepandemic health.

## Teaser

This cohort study examines the association between certain health conditions and lifestyle factors with time to recovery from SARS-CoV-2 infection in a US population-based meta-cohort.

## Introduction

Persistent symptoms and disability following SARS-CoV-2 infection, sometimes termed post–COVID-19 condition (PCC),^1,2^ are of major public health concern. Reports on the epidemiology of recovery from SARS-CoV-2 have varied considerably, depending on outcome definitions,^3,4^ sampling strategies, and phases of the pandemic. Estimates for the proportion of individuals with postacute symptoms lasting 28 days or more have ranged from 17% to 81%.^5,6,7,8^ The estimated impact of vaccination on risk of PCC has varied, with positive^9,10^ and negative^11^ associations in different reports, and there remains limited information on the relative risk of PCC after exposure to the Omicron variant, which rose to dominance in late 2021.

This report describes the epidemiologic features of recovery from SARS-CoV-2 over the first 3 years of the US pandemic period in 14 longstanding cohort studies that included diverse participants from across the US.^12^ Compared with studies leveraging electronic health records,^8,13,14^ our prospective study design includes infections diagnosed at home, which is currently mainstream and recommended practice,^15^ and offers reliable prepandemic measures of preinfection health and lifestyle factors. Using these unique longitudinal data, we investigated temporal trends in, and prepandemic and pandemic-era factors associated with, time to recovery from SARS-CoV-2 infection.

## Methods

### Design

This cohort study uses data from the Collaborative Cohort of Cohorts for COVID-19 Research (C4R), a nationwide prospective meta-cohort of adults comprising 14 established prospective, National Institutes of Health–funded cohort studies (eMethods and eTable 1 in Supplement 1).^12,16,17,18,19,20,21,22,23,24,25,26,27,28,29,30^ The C4R was approved by the institutional review boards at all participating institutions. Cohort participants previously consented to in-person, telephone, and/or email contact and for abstraction of medical records. Additional consent for COVID-19 data collection was obtained according to cohort-specific procedures, including verbal, remote, and traditional written informed consent.^12^ This study followed the Strengthening the Reporting of Observational Studies in Epidemiology (STROBE) reporting guideline.

Starting as early as 1971, cohort investigators have been collecting data on clinical and subclinical diseases and their risk factors among participants from across the US with diverse racial, ethnic, and socioeconomic backgrounds. Participants who were alive and not lost to follow-up on March 1, 2020, were eligible for C4R enrollment. This report includes participants with self-reported nonfatal SARS-CoV-2 infection and information on recovery status ascertained by questionnaires administered from April 1, 2020, through February 28, 2023.

### Infection

The C4R study ascertained SARS-CoV-2 infection using 2 waves of questionnaires administered across the C4R population via telephone interview, mailed pamphlet, electronic survey, and/or in-person examinations (eTables 3 and 4 in Supplement 1).^12,31^ Self-reported infections were confirmed, when possible, via medical records for hospitalizations and deaths linked to SARS-CoV-2 plus a serosurvey (eTable 5 in Supplement 1). The present analysis includes all participants with a history of SARS-CoV-2 infection as defined by self-report in response to a questionnaire, with sensitivity analyses limited to cases confirmed by self-report of a positive test, medical records, or the serosurvey. Acute infection severity was defined by self or proxy report or medical record review (eTables 3-5 in Supplement 1).^32^ Infection date, which was ascertained by questionnaire and confirmed by medical records, where possible, was used to infer the relevant pandemic wave.^33^

Vaccination status at time of infection was defined based on comparison of self-reported infection and vaccination dates. Number of vaccine doses and vaccine manufacturer were self-reported.

Reinfections, defined by self-report or as infections occurring at an interval of at least 6 months, were excluded from primary analyses due to limited data. Selected epidemiologic features were examined in secondary analyses.

### Recovery

Participants were asked, “Following your COVID-19 infection, would you say you are completely recovered from COVID-19 now?” (eTables 3 and 4 in Supplement 1). Participants responding yes were asked, “How long did it take for you to recover?” The response was used to define time to recovery in days. Among participants who had not completely recovered at the time of inquiry, time to nonrecovery was defined as the number of days from infection onset to questionnaire completion, the timing of which was independent of infection history. Nonrecovery at 90 days was classified if time to recovery or time to nonrecovery was 90 days or longer.

### Factors Associated With Recovery

Potential factors associated with time to recovery were selected a priori from data available in most cohorts.^12^ Details on definitions are provided in the eMethods in Supplement 1. Sociodemographic factors were self-reported. Race and ethnicity (including American Indian or Alaska Native, Asian, Hispanic or Latino, non-Hispanic Black, and non-Hispanic White) were examined since they have been associated with disparities in COVID-19 outcomes.^34^ Prepandemic health measures were obtained from the examination closest to the time of C4R enrollment, with a median interval of 5 years (IQR, 4-12 years) (eTable 1 in Supplement 1).^12^ Anthropometry was performed using standardized methods. Health conditions were harmonized from self-reported physician diagnoses, objective measures, and/or questionnaire items using standard clinical and epidemiologic definitions. Diabetes was self-reported or defined by fasting blood glucose of 126 mg/dL or higher or use of hypoglycemic medications. Hypertension was defined by blood pressure of 140/90 mm Hg or higher or use of antihypertensive medications. Estimated glomerular filtration rate was calculated using the Chronic Kidney Disease Epidemiology Collaboration equation. A history of asthma, chronic obstructive pulmonary disease (COPD), emphysema, and chronic bronchitis was self-reported. A history of elevated depressive symptoms was defined by a score of 10 or greater on the 10-item Center for Epidemiological Studies Depression scale. Clinical cardiovascular disease (CVD) was defined as a self-report of myocardial infarction, coronary heart disease, or heart failure or the occurrence of relevant, adjudicated health events over cohort follow-up prior to the pandemic.

### Statistical Analysis

#### Primary Analyses

Analyses of time to recovery used date of infection as the baseline and recovery as the outcome. Participants who reported nonrecovery prior to 90 days were censored at the time of questionnaire. Median time to recovery was plotted according to infection during specific variant waves, stratified by vaccination status at time of infection.

Kaplan-Meier curves were used to estimate probability of nonrecovery by 90 days and restricted mean recovery times; differences in recovery times by the factors were assessed by log-rank test. Cox proportional hazards regression was performed to assess multivariable-adjusted associations with recovery by 90 days. All factors were included in fully adjusted models with 2 exceptions. Acute infection severity was hypothesized to operate as a mediator and, therefore, was included in sensitivity analyses only. Race and ethnicity were collinear with the source cohort and were not included in the main model, which treated cohort as a stratum term. This approach allows each cohort to have its own baseline hazard function, thereby accommodating potential cohort heterogeneity. Effect modification by vaccination status at time of infection was assessed by interaction terms and stratified models.

Mediation of associations by acute infection severity was tested with respect to significant factors identified in the multivariable-adjusted Cox model using a parametric model–based mediation approach.^35^ Multiple imputation was used for missing data (eTable 6 in Supplement 1).^36,37^ A 2-tailed α of .05 was considered statistically significant. Analyses were completed using R, version 4.0.0 (R Foundation for Statistical Computing). Additional details are provided in the eMethods in Supplement 1.

#### Secondary Analyses

Time to recovery from reinfections was investigated, and differences in restricted mean recovery times were assessed for covariates identified as significant in the main models. Associations of 1 vs 2 doses of mRNA vaccine were explored. Cox proportional hazards models were repeated with inclusion of participants who experienced fatal COVID-19, which was treated as nonrecovery at 90 days; participants with asymptomatic COVID-19 infection; restriction to participants with definite SARS-CoV-2 infection based on evidence of confirmatory SARS-CoV-2 testing (eTable 5 in Supplement 1); and participants with imputed recovery time. Models not stratified by cohort were performed to explore associations with race, ethnicity, and cohort.

## Results

### Participants

Of 53 143 eligible participants, 49 319 (92.8%) responded to at least 1 C4R questionnaire, of whom 6980 (14.2%) reported a history of first nonfatal SARS-CoV-2 infection and 5036 of these (72.1%) provided information on time to recovery. Excluding asymptomatic and fatal cases, there were 4708 participants included in the main analysis (eMethods and eTable 2 in Supplement 1). A flowchart illustrating inclusion and exclusion criteria is provided as eFigure 1 in Supplement 1.

The mean (SD) age of the analytic sample was 61.3 (13.8) years in 2020; 2952 participants (62.7%) were women and 1756 (37.3%) men; and the distribution of race and ethnicity was 371 American Indian or Alaska Native (7.9%), 50 Asian (1.1%), 2086 Hispanic or Latino (44.3%), 622 non-Hispanic Black (13.2%), and 1578 non-Hispanic White (33.5%) (Table 1). Included participants were younger and had fewer comorbidities compared with participants who were excluded due to missing data on recovery (eTable 7 in Supplement 1); nonetheless, the distribution of characteristics was similar.

**Table 1.** Characteristics of C4R Participants According to Recovery Status at 90 Days Post Infection

Characteristic	Recovery status, No. (%)^a^			
	Observed recovery by 90 d	Nonrecovery censored at <90 d	Nonrecovery by 90 d	Total
No. of participants	3532 (75.0)	334 (7.1)	842 (17.9)	4708 (100)
Age in March 2020, y				
<50	705 (20.0)	65 (19.4)	171 (20.4)	941 (20.0)
50-64	1557 (44.1)	121 (36.3)	354 (42.0)	2032 (43.2)
65-79	988 (28.0)	108 (32.5)	253 (30.0)	1350 (28.7)
≥80	282 (8.0)	40 (11.9)	64 (7.6)	386 (8.2)
Sex				
Female	2157 (61.1)	225 (67.4)	570 (67.7)	2952 (62.7)
Male	1375 (38.9)	109 (32.6)	272 (32.3)	1756 (37.3)
Race and ethnicity				
American Indian or Alaska Native	202 (5.7)	25 (7.5)	144 (17.1)	371 (7.9)
Asian	39 (1.1)	7 (2.1)	4 (0.5)	50 (1.1)
Black, non-Hispanic	491 (13.9)	43 (12.9)	88 (10.5)	622 (13.2)
Hispanic or Latino	1642 (46.5)	112 (33.5)	332 (39.4)	2086 (44.3)
White, non-Hispanic	1157 (32.8)	147 (44.0)	274 (32.5)	1578 (33.5)
Educational attainment				
Less than high school	534 (15.1)	35 (10.5)	118 (14.0)	687 (14.6)
High school	845 (23.9)	81 (24.3)	219 (26.0)	1146 (24.3)
Some college	630 (17.8)	57 (17.0)	184 (21.8)	871 (18.5)
College degree	1523 (43.1)	161 (48.2)	321 (38.1)	2005 (42.6)
Smoking status				
Never	2069 (58.6)	144 (43.1)	405 (48.1)	2618 (55.6)
Former	1051 (29.8)	128 (38.3)	279 (33.1)	1458 (31.0)
Current	412 (11.7)	62 (18.6)	158 (18.8)	633 (13.4)
Body mass index^b^				
<25	721 (20.4)	80 (24.0)	160 (19.0)	961 (20.4)
25 to <30	1351 (38.3)	107 (32.0)	270 (32.0)	1728 (36.7)
≥30	1460 (41.3)	147 (44.0)	412 (49.0)	2020 (42.9)
Diabetes				
Absent	2918 (82.6)	263 (78.8)	653 (77.6)	3834 (81.4)
Present	614 (17.4)	71 (21.2)	189 (22.4)	874 (18.6)
Hypertension				
Absent	2241 (63.4)	189 (56.5)	460 (54.6)	2889 (61.4)
Present	1291 (36.6)	145 (43.5)	383 (45.4)	1819 (38.6)
CVD				
Absent	3275 (92.7)	298 (89.3)	746 (88.6)	4319 (91.7)
Present	258 (7.3)	36 (10.7)	96 (11.4)	389 (8.3)
eGFR <45 mL/min/1.73 m^2^				
Absent	3427 (97.0)	321 (96.0)	811 (96.6)	4561 (96.9)
Present	105 (3.0)	13 (4.0)	29 (3.4)	147 (3.1)
Asthma				
Absent	2968 (84.0)	271 (81.0)	682 (81.0)	3921 (83.3)
Present	564 (16.0)	64 (19.0)	160 (19.0)	787 (16.7)
COPD				
Absent	3315 (93.9)	288 (86.3)	742 (88.1)	4345 (92.3)
Present	217 (6.1)	46 (13.7)	100 (11.9)	363 (7.7)
Elevated depressive symptoms				
Absent	2861 (81.0)	260 (77.8)	582 (69.1)	3702 (78.6)
Present	671 (19.0)	74 (22.2)	261 (30.9)	1006 (21.4)
Vaccination prior to infection				
No (unvaccinated)	2771 (78.5)	211 (63.3)	760 (90.2)	3743 (79.5)
Yes (vaccinated)	761 (21.5)	123 (36.7)	82 (9.8)	966 (20.5)
Infection wave				
First (wild type)	777 (22.0)	68 (20.4)	179 (21.3)	1025 (21.8)
Second (wild type)	454 (12.8)	32 (9.6)	107 (12.7)	593 (12.6)
Third (Alpha)	1147 (32.5)	57 (17.1)	371 (44.1)	1578 (33.5)
Fourth (Alpha)	222 (6.3)	15 (4.5)	55 (6.5)	292 (6.2)
Fifth (Delta)	447 (12.6)	67 (20.1)	85 (10.1)	598 (12.7)
Sixth (Omicron)	485 (13.7)	95 (28.4)	45 (5.3)	625 (13.4)
Acute infection severity				
Outpatient	3173 (88.3)	295 (88.3)	643 (76.4)	4111 (87.3)
Noncritical hospitalized	279 (7.9)	28 (8.4)	142 (16.9)	449 (9.5)
Critical hospitalized	80 (2.3)	11 (3.3)	57 (6.8)	148 (3.1)
Source cohort				
ARIC	201 (5.8)	27 (8.4)	29 (3.9)	257 (5.7)
CARDIA	274 (7.9)	8 (2.5)	29 (3.9)	311 (6.9)
COPDGene	116 (3.4)	84 (26.2)	117 (15.6)	317 (7.0)
FHS	351 (9.9)	38 (11.4)	58 (6.9)	447 (9.5)
HCHS/SOL	1498 (43.4)	101 (31.5)	302 (40.2)	1901 (42.0)
JHS	165 (4.8)	6 (1.9)	14 (1.9)	185 (4.1)
MASALA	23 (0.7)	5 (1.6)	3 (0.4)	31 (0.7)
MESA	164 (4.8)	12 (3.7)	37 (4.9)	213 (4.7)
NOMAS	97 (2.8)	9 (2.8)	24 (3.2)	130 (2.9)
PrePF	75 (2.2)	3 (0.9)	12 (1.6)	90 (2.0)
REGARDS	270 (7.8)	6 (1.9)	61 (8.1)	337 (7.5)
SARP	31 (0.9)	3 (0.9)	8 (1.1)	42 (0.9)
SHS	195 (5.5)	25 (7.5)	141 (16.7)	361 (7.7)
SPIROMICS	72 (2.1)	7 (2.2)	7 (0.9)	86 (1.9)

Abbreviations: ARIC, Atherosclerosis Risk in Communities; C4R, Collaborative Cohort of Cohorts for COVID-19 Research; CARDIA, Coronary Artery Risk Development in Young Adults; COPD, chronic obstructive pulmonary disease; COPDGene, COPD Gene Study; CVD, cardiovascular disease; eGFR, estimated glomerular filtration rate; FHS, Framingham Heart Study; HCHS/SOL, Hispanic Community Health Study/Study of Latinos; JHS, Jackson Heart Study; MASALA, Mediators of Atherosclerosis of South Asians Living in America; MESA, Multi-Ethnic Study of Atherosclerosis; NOMAS, Northern Manhattan Study; PrePF, Preventing Pulmonary Fibrosis; REGARDS, Reasons for Geographic and Racial Differences in Stroke; SARP, Severe Asthma Research Program; SHS, Strong Heart Study; SPIROMICS, Subpopulations and Intermediate Outcome Measures in COPD Study.

^a^ This table presents data based on the average of 10 multiply imputed datasets. Numbers may not sum exactly to totals due to rounding. Column percentages are reported.

^b^ Measured by weight in kilograms divided by height in meters squared.

**Figure 1.**
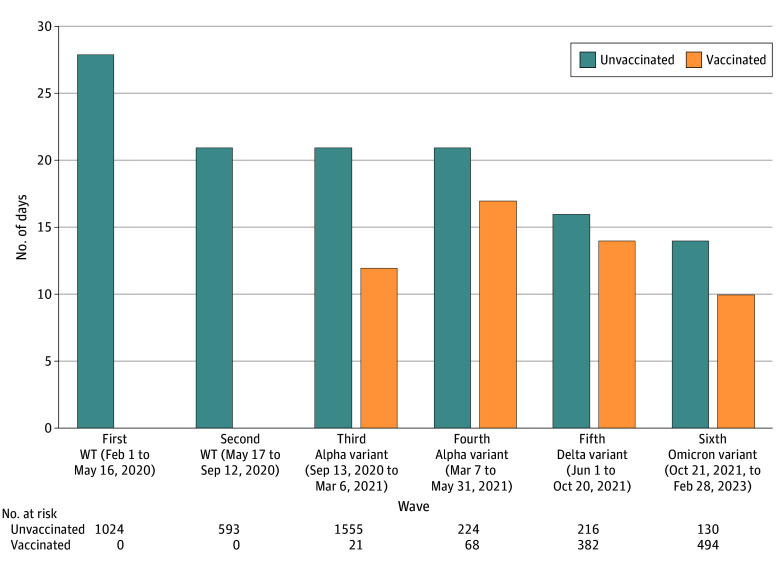
Trends in Median Time to Recovery After SARS-CoV-2 by Vaccination Status at Time of Infection WT indicates wild type.

Infections occurred across 6 pandemic waves (Figure 1). Hospitalized participants numbered 597 (12.6%), and 148 (3.1%) required critical care. There were 3825 confirmed infections (81.2%) (eTable 5 in Supplement 1). Vaccination prior to infection was reported by 966 participants (20.5%), of whom 57 (5.9%) had received only 1 mRNA vaccine dose.

### Time to Recovery

At the time of questionnaire completion (mean [SD] time since infection, 250 [201] days), 3656 participants (77.6%) reported that they had completely recovered. Of 1052 who had not completely recovered, 334 (31.7%) completed questionnaires less than 90 days post infection; hence, recovery status at 90 days was not defined.

Median time to recovery was 20 days (IQR, 8-75 days) and decreased over time (Figure 1). Participants who were vaccinated at the time of infection had shorter median time to recovery. Probability of nonrecovery by 90 days was 22.5% (95% CI, 21.2%-23.7%) and differed for the pre-Omicron (23.3%; 95% CI, 22.0%-24.6%) vs Omicron (16.8%; 95% CI, 13.3%-20.2%) waves.

**Figure 2.**
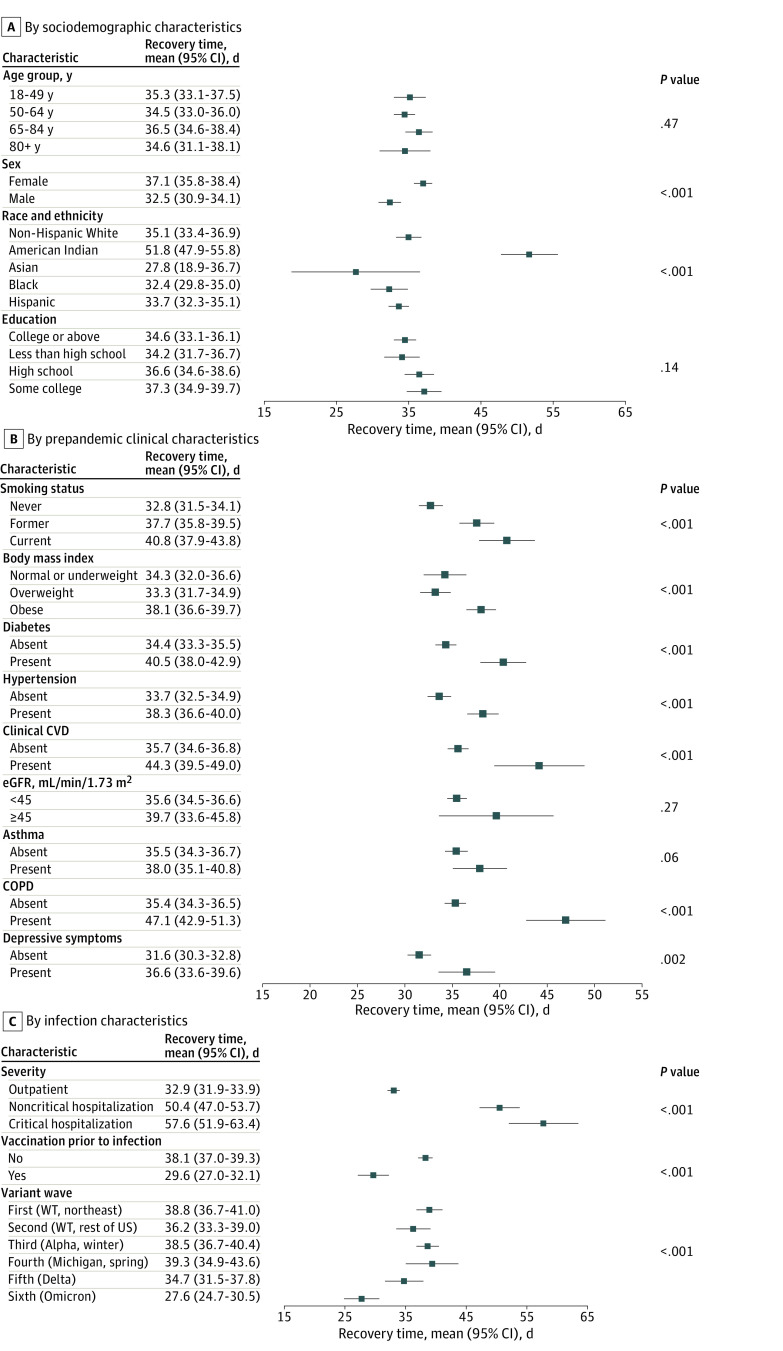
Unadjusted Restricted Mean Recovery Time From SARS-CoV-2 Infection Restricted mean recovery time was calculated from the unadjusted Kaplan-Meier curve for each characteristic, censored at 90 days post infection. Characteristics of participants who had been observed to recover by 90 days were compared with those who had not recovered, or whose follow-up was censored prior to 90 days, by log-rank test. COPD indicates chronic obstructive pulmonary disease; CVD, cardiovascular disease; eGFR, estimated glomerular filtration rate; WT, wild type.

Restricted mean recovery time was 35.4 days (95% CI, 34.4-36.4 days) and was associated with sociodemographic, clinical, lifestyle, and infection-related factors (Figure 2). Pertinent negatives included lack of significant differences by age group, educational attainment, or prepandemic chronic kidney disease or asthma. Mean recovery time was 57.6 days (95% CI, 51.9-63.3 days) after critical hospitalization vs 32.9 days (95% CI, 31.9-33.9 days) for outpatient infection.

Multivariable-adjusted hazard ratios (HRs) for recovery by 90 days are presented in Table 2. Improved (greater) hazard of recovery was associated with vaccination prior to infection (HR, 1.30; 95% CI, 1.11-1.51) and infection during the Omicron wave (HR, 1.25; 95% CI, 1.06-1.49). Lesser recovery was associated with female vs male sex (HR, 0.85; 95% CI, 0.79-0.92) and clinical CVD vs no CVD (HR, 0.84; 95% CI, 0.71-0.99). Although not significant, lesser recovery was found for obesity vs normal or underweight (HR, 0.91; 95% CI, 0.82-1.00) and COPD vs no COPD (HR, 0.88; 95% CI, 0.75-1.03). Recovery was not associated with age group; educational attainment; or prepandemic smoking, diabetes, hypertension, chronic kidney disease, asthma, or elevated depressive symptoms.

**Table 2.** Factors Associated With Recovery by 90 Days After SARS-CoV-2 Infection, With Multivariable Adjustment

Variable	HR (95% CI)^a^	*P* value
Age, y		
<50	1 [Reference]	NA
50-64	1.04 (0.95-1.15)	.41
65-79	1.04 (0.92-1.17)	.55
≥80	1.01 (0.84-1.22)	.91
Sex		
Male	1 [Reference]	NA
Female	0.85 (0.79-0.92)	<.001
Educational attainment		
College	1 [Reference]	NA
<High school	1.05 (0.94-1.17)	.41
High school	1.04 (0.95-1.14)	.38
Some college	1.02 (0.92-1.12)	.72
Smoking status		
Never	1 [Reference]	NA
Former	0.95 (0.88-1.03)	.20
Current	0.92 (0.82-1.03)	.13
Body mass index^b^		
<25	1 [Reference]	NA
25 to <30	1.01 (0.92-1.11)	.84
≥30	0.91 (0.82-1.00)	.051
Diabetes		
Absent	1 [Reference]	NA
Present	0.94 (0.85-1.03)	.19
Hypertension		
Absent	1 [Reference]	NA
Present	0.95 (0.88-1.03)	.24
Cardiovascular disease		
Absent	1 [Reference]	NA
Present	0.84 (0.71-0.99)	.045
eGFR, mL/min/1.73 m^2^		
≥45	1 [Reference]	NA
<45	0.90 (0.73-1.11)	.31
Asthma		
Absent	1 [Reference]	NA
Present	0.99 (0.90-1.10)	.91
COPD		
Absent	1 [Reference]	
Present	0.88 (0.75-1.03)	.10
Elevated depressive symptoms		
Absent	1 [Reference]	NA
Present	0.92 (0.82-1.03)	.14
Vaccination prior to infection		
No	1 [Reference]	NA
Yes	1.30 (1.11-1.51)	.001
Infection wave		
First (WT, February 1 to May 16, 2020)	1 [Reference]	NA
Second (WT, May 17 to September 12, 2020)	1.05 (0.93-1.19)	.41
Third (Alpha variant, September 13, 2020, to March 6, 2021)	0.98 (0.89-1.08)	.66
Fourth (Alpha variant, March 7 to May 31, 2021)	0.93 (0.79-1.10)	.38
Fifth (Delta variant, June 1 to October 20, 2021)	0.98 (0.84-1.15)	.83
Sixth (Omicron variant, October 21, 2021, to February 28, 2023)	1.25 (1.06-1.49)	.01

Abbreviations: COPD, chronic obstructive pulmonary disease; eGFR, estimated glomerular filtration rate; HR, hazard ratio; NA, not applicable; WT, wild type.

^a^ Cox proportional hazards models were estimated to assess associations of time-to-recovery with the factors of interest. Estimates were generated from models adjusted for all the factors listed in the table. Hazards ratios greater than 1 indicate faster recovery, whereas HRs less than 1 indicate slower recovery.

^b^ Measured by weight in kilograms divided by height in meters squared.

Models stratified by vaccination status yielded fewer associations in vaccinated (n = 966) vs unvaccinated (n = 3743) participants (eFigure 2 in Supplement 1). Women showed unfavorable recovery in both groups. Prepandemic clinical CVD was unfavorably associated in unvaccinated (HR, 0.84; 95% CI, 0.69-0.99) but not vaccinated (HR, 0.94; 95% CI, 0.63-1.42) participants. Infection during the Omicron wave was favorably associated with recovery in unvaccinated (HR, 1.32; 95% CI, 1.05-1.66) but not vaccinated (HR, 1.23; 95% CI, 0.71-2.12) participants. Nonetheless, there were no significant multiplicative interactions with vaccination status for any factor.

### Infection Severity

When added to the multivariable model, infection severity was strongly associated with recovery (eTable 8 in Supplement 1). Compared with outpatient infection, HRs for recovery were 0.59 for noncritical hospitalization (95% CI, 0.52-0.67) and 0.46 for critical illness hospitalization (95% CI, 0.36-0.57). Other effect estimates were comparable to the main model.

Favorable associations of recovery with vaccination status (33.4%; *P* = .001) and infection during the Omicron wave (17.6%; *P* = .01) were partially mediated by associations with reduced infection severity (Table 3). The unfavorable association of recovery with clinical CVD was partially mediated by its association with greater severity (20.0%; *P* = .047). There was significant negative mediation of sex effects (−24.3%; *P* = .006): Compared with women, the greater risk of severe acute illness observed in men offset the shorter time to recovery in men.

**Table 3.** Mediation of Associations With Time to Recovery via Severity of Acute SARS-CoV-2 Infection

Factor	Severity of acute SARS-CoV-2 infection to time to recovery, No. (%)			Mediation analysis, mean days to recovery (95% CI)^a^			Proportion of effect mediated by infection severity, % (*P* value)
	Outpatient	Noncritical hospitalization	Critical hospitalization	Average mediated effect	Average direct effect	Total effect	
Vaccination status at time of infection							
Unvaccinated	3191 (85.3)	420 (11.2)	132 (3.5)	1 [Reference]	1 [Reference]	1 [Reference]	NA
Vaccinated	920 (95.3)	29 (3.0)	17 (1.7)	−5.8 (−9.4 to −3.5)	−11.8 (−21.6 to −1.9)	−17.5 (−27.2 to −7.9)	33.4 (.001)
Variant wave of infection							
Pre Omicron	3508 (85.9)	440 (10.8)	135 (3.3)	1 [Reference]	1 [Reference]	1 [Reference]	NA
Omicron	603 (96.5)	9 (1.4)	13 (2.1)	−2.9 (−5.6 to −0.2)	−13.6 (−21.4 to −5.7)	−16.4 (−24.5 to −8.4)	17.6 (.01)
Sex							
Male	1531 (85.4)	186 (10.4)	75 (4.2)	1 [Reference]	1 [Reference]	1 [Reference]	NA
Female	2580 (88.5)	263 (9.0)	73 (2.5)	−2.4 (−4.6 to 0.3)	12.5 (7.4 to 17.7)	10.1 (4.5 to 15.7)	−24.3 (.006)
CVD							
Absent	3820 (88.5)	376 (8.7)	122 (2.8)	1 [Reference]	1 [Reference]	1 [Reference]	NA
Present	291 (74.8)	73 (18.7)	26 (6.6)	2.0 (−0.6 to 7.0)	11.6 (1.9 to 25.0)	14.5 (0.0 to 29.0)	20.0 (.047)

Abbreviations: CVD, cardiovascular disease; NA, not applicable.

^a^ Mediation of associations by severity of acute infection was tested with respect to significant factors identified in the multivariable-adjusted Cox proportional hazards model. Since there are no standard methods to assess mediation within the Cox proportional hazards model framework, we applied a parametric model–based mediation analysis to estimate the mean mediation effects, the mean direct effects, and the mean percent mediated. Recovery by 90 days was modeled using an accelerated failure time model with a Weibull distribution, and ordinal logistic regression was used to model infection severity.

### Secondary Analyses

Median time to recovery among 212 reinfections was 27 days (IQR, 9-90 days), and estimated probability of nonrecovery by 90 days was 24.4% (95% CI, 19.2%-31.0%). Greater restricted mean recovery time after reinfection was seen in women compared with men (42.3 vs 31.5 days; *P* = .03) and in pre-Omicron compared with Omicron infections (39.8 vs 28.6 days; *P* = .09). There was no significant association with CVD or vaccination status (eTable 9 in Supplement 1). Compared with receipt of at least 2 doses of mRNA vaccine, receipt of a single dose was not significantly associated with recovery by 90 days (adjusted HR, 0.89; 95% CI, 0.64-1.24).

Results were similar with inclusion of fatal and asymptomatic COVID-19 cases (eTable 10 in Supplement 1) and with exclusion of nonconfirmed infections (eTable 11 in Supplement 1). In models that included cohort as a factor rather than a stratum term, there were significant associations observed for the 2 cohorts with the highest restricted mean recovery times (Genetic Epidemiology of COPD study, 61.9 days; Strong Heart Study, 52.1 days) (eTable 12 and eFigure 3 in Supplement 1). Models excluding these cohorts yielded similar results to the main model, as did models excluding the 4 cohorts with lung disease (eTable 13 in Supplement 1).

In models that did not account for cohort effects (eTable 12 in Supplement 1), American Indian or Alaska Native participants showed adverse associations with recovery compared with non-Hispanic White participants (HR, 0.64; 95% CI, 0.53-0.78). This association was not mediated by infection severity, although the sample size was limited (371 participants). In these models, significant associations were also observed for smoking vs never smoking (former smoking: HR, 0.89 [95% CI, 0.82-0.96]; current smoking: HR, 0.82 [95% CI, 0.74-0.92]) and COPD vs no COPD (HR, 0.71; 95% CI, 0.61-0.83).

## Discussion

In a large, racially and ethnically diverse, US population-based meta-cohort with standardized, prospective data collection during the pandemic, more than 1 in 5 participants did not recover from SARS-CoV-2 infection by 90 days post infection.^3^ Self-reported recovery by 90 days was less likely in women than men and in participants with vs without prepandemic clinical CVD. Recovery was favorably associated with vaccination prior to infection and infection during the Omicron wave, and these associations were partially mediated by reduced severity of acute infection.

Our findings are consistent with a substantial US population burden of PCC. The Researching COVID to Enhance Recovery (RECOVER) initiative—a large, prospective, case-control study of PCC—defined a framework to classify PCC based on self-report of 12 symptoms at 6 months post infection^38^ and found a 10% prevalence of symptom score–defined PCC at 6-month follow-up, with fewer PCC-like symptoms following Omicron vs pre-Omicron infections. The C4R did not collect the same symptom data as RECOVER; hence, we cannot cross validate the RECOVER score using C4R’s prospective population-based design. Furthermore, we examined recovery over a 3-month postinfection interval for consistency with the World Health Organization definition of PCC^3^ and to reduce loss to follow-up. Nonetheless, our main findings are in general agreement. It will be important to examine longer-term trajectories of recovery and to distinguish COVID-19–related from non–COVID-19 causes of PCC-like symptoms using data being collected by C4R via a third administration of questionnaires, which were harmonized with RECOVER questionnaires to support complementary research and opportunities for cross validation.

Our observations regarding reduction in the burden of PCC over time are consistent with other reports and may be partly due to reductions in the risk of severe SARS-CoV-2 illness over the course of the pandemic.^39^ Participants with infections during the Omicron wave showed shorter recovery times, consistent with findings from RECOVER.^38^ Our finding of an only 17.6% mediation of Omicron effects by severity may speak to the reduced pathogenicity of Omicron vs prior variants.^40^ Our models suggest that the association of vaccination with reduced recovery time was 33.4% mediated by reduced infection severity. Although vaccination status at the time of infection was not confirmed to modify associations of recovery with any of the factors, the effect estimate for clinical CVD was substantially attenuated among vaccinated vs unvaccinated participants. Our findings support the use of vaccines to reduce the risk of long COVID, particularly in high-risk groups.

Longer recovery times were observed in participants with prepandemic health conditions. With simultaneous adjustment for all prepandemic conditions, only clinical CVD was associated with recovery; infection severity mediated this association by 20.0%, suggesting the importance of alternative pathways, such as systemic inflammation or endotheliopathy.^41^ Nonsignificant multivariable-adjusted findings for other conditions could be influenced by collinearity in the setting of multimorbidity. Because some participants may have developed health conditions before SARS-CoV-2 infection but after the most recent cohort examination, effect estimates for these conditions were presumably conservative. Differential response rates by severity of infection and general health may also have blunted associations; participants included in the analysis appeared healthier than those excluded due to missing outcome data. Overall, C4R may be characterized by healthy participant bias relative to the general population. Recovery time in the Genetic Epidemiology of COPD cohort, which follows patients with COPD across the spectrum of clinical severity, was substantially longer than in other cohorts. Sensitivity analyses that were not stratified by cohort suggested additional significant multivariable-adjusted associations with smoking history and COPD.

Unfavorable recovery outcomes were observed in American Indian or Alaska Native participants in models that did not account for cohort. Consistent with the disproportionate impact of the COVID-19 pandemic on US Indigenous communities,^42,43^ American Indian or Alaska Native participants reported a higher burden of severe infection and nonrecovery at 90 days. Additional research is needed on the extent to which the pandemic may have exacerbated US social and health disparities, including among Indigenous communities. Inclusion of racially and ethnically diverse participants in PCC mechanistic research and clinical trials remains essential to identifying and equitably distributing interventions.

Consistent with prior reports,^44,45,46,47^ women experienced worse recovery despite a lower rate of severe acute illness. This finding could be due to a reporting bias differential by sex, although other possibilities must be considered. Sex differences in risk of PCC, and particularly PCC subphenotypes characterized by neurologic, musculoskeletal, and autoimmune conditions,^48^ could be explained by multiple mechanisms, including differences in the immune response and higher risk of autoreactivity and thrombosis in women (vs men),^49^ that merit further study.

### Limitations

Limitations include the potential for measurement error, uncontrolled confounding, and selection bias. Infections were primarily self-reported; however, results among participants with confirmed infections were similar. Recovery time was self-reported, and research is needed using objective biological and physiologic measures, particularly for longitudinal comparisons with prepandemic measurements. Data were limited on recovery following reinfection,^50^ yet secondary analyses suggested that the recovery features of reinfections and incident infections were similar. Questionnaires did not assess development of new symptoms after infection, which has been reported^3^; however, acutely asymptomatic cases with nonzero recovery time were included in sensitivity analyses, suggesting that participants with postacute emergence of symptoms may be represented. Data from 2022 to 2023 were relatively limited; ongoing C4R follow-up will provide additional information in the setting of Omicron variant dominance, availability of antiviral therapies (eg, nirmatrelvir/ritonavir), and repeated vaccination. The nonsignificant comparison of 1 vs 2 or more mRNA vaccine doses in this study was underpowered. Data not missing at random could introduce bias. Because of the potential for type I error due to multiple comparisons, sensitivity analyses should be interpreted as exploratory. Most cohorts did not include population-representative sampling, limiting generalizability. Pooling across cohorts introduces heterogeneity, yet COVID-19–related data collection was standardized, prepandemic measures were harmonized, and models were stratified by cohort. Sensitivity analyses that did not account for cohort were consistent with the main findings, although several additional significant associations were observed, suggesting that our approach was conservative.^12,51^

## Conclusions

This cohort study found that 1 in 5 adults infected with SARS-CoV-2 did not fully recover by 3 months post infection in a racially and ethnically diverse US population-based sample. Recovery by 90 days was less likely in women and participants with prepandemic clinical CVD. Vaccination prior to infection and infection during the Omicron variant wave were associated with greater recovery, which was partially mediated by reduced acute infection severity. Results were similar for reinfections. Further investigation on the longer-term prognosis and mechanisms of PCC, including comparisons of multiorgan structure and function before and after infection, is critical to inform treatment and prevention.

## Notes

Supplement 1.
eMethods.
**eTable 1.** Characteristics of Participants in C4R Cohorts, United States, March 1, 2020 **eTable 2.** Questionnaire Administration, Including Response Rates and Availability of Complete Data for Analysis, by Cohort **eTable 3.** Definition of Infection, Infection Severity, and Time to Recovery Via C4R Wave 1 Questionnaires, Administered April 1, 2020, Through May 29, 2022 **eTable 4.** Definition of Infection, Infection Severity, and Time to Recovery Via C4R Wave 2 Questionnaires, Administered March 1, 2021, Through February 28, 2023 **eTable 5.** Classification of Selected COVID-19 Outcomes Available in C4R as Confirmed vs Probably, and Number (Percent) of Cases in the Analytic Sample (n = 4708) **eTable 6.** Missingness of Covariate Data Among Participants Included in the Analysis **eTable 7.** Comparison of Characteristics of Infected C4R Participants Included vs Not Included in the Analytic Sample **eTable 8.** Correlates of Recovery by 90 Days After SARS-CoV-2 Infection After Multivariable Adjustment, Adjusted for Disease Severity **eTable 9.** Restricted Mean Recovery Time From Reinfections (n = 212) in Strata of Covariates Identified in the Main Model **eTable 10.** Main Correlates of Recovery in Time to Event Models, Including Cases of Asymptomatic or Fatal SARS-CoV-2 Infection **eTable 11.** Main Correlates of Recovery in Time to Event Models, After Exclusion of Probable (Nondefinite) Cases **eTable 12.** Main Correlates of Recovery in Time to Event Models, Without Stratification by Cohort, Adjusting for Race, Ethnicity, and Cohort **eTable 13.** Main Correlates of Recovery in Time to Event Models, After Exclusion of Selected Cohorts **eFigure 1.** CONSORT Diagram of Participants Included in Analyses **eFigure 2.** Correlates of Recovery by 90 Days After SARS-CoV-2 Infection, After Multivariable Adjustment, Stratified by Vaccination Status at Time of Infection **eFigure 3.** Restricted Mean Recovery Time in Days Following SARS-CoV-2 Infection, by Cohort
eReferences

Supplement 2.
Data Sharing Statement
