# Error in Figure 2

**DOI:** 10.1001/jamanetworkopen.2024.27640

**Published:** 2024-07-17

In the Original Investigation titled “Epidemiologic Features of Recovery From SARS-CoV-2 Infection,”^1^ published June 17, 2024, in Figure 2C, the recovery time data for noncritical hospitalization and for the fourth variant wave (Michigan, spring) were incorrect. The correct value for noncritical hospitalization is 50.4 days (95% CI, 47.0-53.7 days), and the correct value for the fourth variant wave is 39.3 days (95% CI, 34.9-43.6) days. This article has been corrected.^1^
