# Supplementary material for: Epidemiologic Features of Recovery From SARS-CoV-2 Infection
Source: JAMA Netw Open. 2024 Jun 17;7(6):e2417440. doi: 10.1001/jamanetworkopen.2024.17440 (PMC11184459; doi:10.1001/jamanetworkopen.2024.17440)
Supplement: Supplement 1. — eMethods. eTable 1. Characteristics of Participants in C4R Cohorts, United States, March 1, 2020 eTable 2. Questionnaire Administration, Including Response Rates and Availability of Complete Data for Analysis, by Cohort eTable 3. Definition of Infection, Infection Severity, and Time to Recovery Via C4R Wave 1 Questionnaires, Administered April 1, 2020, Through May 29, 2022 eTable 4. Definition of Infection, Infection Severity, and Time to Recovery Via C4R Wave 2 Questionnaires, Administered March 1, 2021, Through February 28, 2023 eTable 5. Classification of Selected COVID-19 Outcomes Available in C4R as Confirmed vs Probably, and Number (Percent) of Cases in the Analytic Sample (n = 4708) eTable 6. Missingness of Covariate Data Among Participants Included in the Analysis eTable 7. Comparison of Characteristics of Infected C4R Participants Included vs Not Included in the Analytic Sample eTable 8. Correlates of Recovery by 90 Days After SARS-CoV-2 Infection After Multivariable Adjustment, Adjusted for Disease Severity eTable 9. Restricted Mean Recovery Time From Reinfections (n = 212) in Strata of Covariates Identified in the Main Model eTable 10. Main Correlates of Recovery in Time to Event Models, Including Cases of Asymptomatic or Fatal SARS-CoV-2 Infection eTable 11. Main Correlates of Recovery in Time to Event Models, After Exclusion of Probable (Nondefinite) Cases eTable 12. Main Correlates of Recovery in Time to Event Models, Without Stratification by Cohort, Adjusting for Race, Ethnicity, and Cohort eTable 13. Main Correlates of Recovery in Time to Event Models, After Exclusion of Selected Cohorts eFigure 1. CONSORT Diagram of Participants Included in Analyses eFigure 2. Correlates of Recovery by 90 Days After SARS-CoV-2 Infection, After Multivariable Adjustment, Stratified by Vaccination Status at Time of Infection eFigure 3. Restricted Mean Recovery Time in Days Following SARS-CoV-2 Infection, by Cohort eReferences [file jamanetwopen-e2417440-s001.pdf]

Supplemental Online Content

Oelsner EC, Sun Y, Balte PP, et al. Epidemiologic features of recovery from SARS-CoV-2 infection in the US. *JAMA Netw Open*. 2024;7(6):e2417440. doi:10.1001/jamanetworkopen.2024.17440

eMethods.

eTable 1. Characteristics of Participants in C4R Cohorts, United States, March 1, 2020

eTable 2. Questionnaire Administration, Including Response Rates and Availability of Complete Data for Analysis, by Cohort

eTable 3. Definition of Infection, Infection Severity, and Time to Recovery Via C4R Wave 1 Questionnaires, Administered April 1, 2020, Through May 29, 2022

eTable 4. Definition of Infection, Infection Severity, and Time to Recovery Via C4R Wave 2 Questionnaires, Administered March 1, 2021, Through February 28, 2023

eTable 5. Classification of Selected COVID-19 Outcomes Available in C4R as Confirmed vs Probably, and Number (Percent) of Cases in the Analytic Sample (n = 4708)

eTable 6. Missingness of Covariate Data Among Participants Included in the Analysis

eTable 7. Comparison of Characteristics of Infected C4R Participants Included vs Not Included in the Analytic Sample

eTable 8. Correlates of Recovery by 90 Days After SARS-CoV-2 Infection After Multivariable Adjustment, Adjusted for Disease Severity

eTable 9. Restricted Mean Recovery Time From Reinfections (n = 212) in Strata of Covariates Identified in the Main Model

eTable 10. Main Correlates of Recovery in Time to Event Models, Including Cases of Asymptomatic or Fatal SARS-CoV-2 Infection

eTable 11. Main Correlates of Recovery in Time to Event Models, After Exclusion of Probable (Nondefinite) Cases

eTable 12. Main Correlates of Recovery in Time to Event Models, Without Stratification by Cohort, Adjusting for Race, Ethnicity, and Cohort

eTable 13. Main Correlates of Recovery in Time to Event Models, After Exclusion of Selected Cohorts

eFigure 1. CONSORT Diagram of Participants Included in Analyses

eFigure 2. Correlates of Recovery by 90 Days After SARS-CoV-2 Infection, After Multivariable Adjustment, Stratified by Vaccination Status at Time of Infection

eFigure 3. Restricted Mean Recovery Time in Days Following SARS-CoV-2 Infection, by Cohort

eReferences

This supplemental material has been provided by the authors to give readers additional information about their work.

© 2024 Oelsner EC et al. *JAMA Network Open.*

eMethods

Cohort Descriptions

Atherosclerosis Risk in Communities (ARIC)[1]: The ARIC study began in the mid 1980s with initial aims for its cohort component being to describe the presence of subclinical atherosclerosis (mainly via carotid ultrasound), the progression of atherosclerosis to clinical cardiovascular disease (CVD), and the association of novel risk factors with CVD. ARIC recruited its cohort of 15,792 men and women aged 45-64 in 1987-89 from four communities: Forsyth County, NC; Jackson, MS; suburban Minneapolis, MN; and Washington County, MD. The investigators used probability sampling to obtain a community wide sample, exclusively sampling African Americans in Jackson and oversampling African Americans in Forsyth County. ARIC conducted a baseline examination of cohort participants and up to eight subsequent examinations prior to the pandemic, with additional follow-up ongoing; performed annual or semi-annual telephone follow-up interviews; and throughout has identified and validated incident CVD and other outcomes, particularly cognitive decline in recent years.

Coronary Artery Risk Development in Young Adults (CARDIA)[2]: CARDIA is a study examining the development and determinants of clinical and subclinical CVD and their risk factors. It began in 1985-1986 with a cohort of 5115 Black and White men and women aged 18-30 years. The participants were selected so that there would be approximately the same number of people in subgroups of race (Black and White), gender (women and men), education (high school or less and more than high school) and age (18-24 and 25-30 years) in each of 4 field centers: Birmingham, AL; Chicago, IL; Minneapolis, MN; and Oakland, CA. These same participants were asked to participate in follow-up examinations during 1987-1988 (Year 2), 1990-1991 (Year 5), 1992-1993 (Year 7), 1995-1996 (Year 10), 2000-2001 (Year 15), 2005-2006 (Year 20), 2010-2011 (Year 25), 2015-2016 (Year 30), and 2020-2022 (Year 35). A majority of the group has been examined at each of the follow-up examinations (91%, 86%, 81%, 79%, 74%, 72%, 72%, 71%, and 67% [despite the impact of the COVID-19 pandemic on Year 35], respectively). While the specific aims of each examination have varied, data have been collected on a variety of factors believed to be related to heart disease. These include conditions with clear links to heart disease such as blood pressure, cholesterol and other lipids, and glucose. Data have also been collected on physical measurements such as weight and body composition as well as lifestyle factors such as dietary and exercise patterns, substance use (tobacco and alcohol), behavioral and psychological variables, medical and family history, and other chemistries (e.g., insulin). In addition, subclinical atherosclerosis has been measured via echocardiography during Years 5, 10, 25, and 30, a chest CT scan during Years 15, 20, 25, and 35, an abdominal CT scan during Years 25 and 35, and carotid ultrasound during Year 20. A brain MRI was performed on a subset of participants at Years 25, 30, and 35. The CARDIA cohort, born between 1955 and 1968, has been influenced substantially by the obesity epidemic at ages younger than participants in other established NHLBI cohorts. Further investigation of the mechanisms linking obesity to derangements in cardiovascular structure and function and the etiology of clinical events promises to generate important new knowledge to inform health promotion and disease prevention efforts.

Genetic Epidemiology of COPD (COPDGene)[3]: COPDGene is a non-interventional, multicenter, longitudinal, case-control study at 21 US sites of smokers with a ≥10 pack-year history with and without COPD and healthy never smokers. The goal was to characterize disease-related phenotypes and explore associations with susceptibility genes. COPDGene research participants were extensively phenotyped with the use of comprehensive symptom and comorbidity questionnaires,

spirometry, chest CT scans, and genetic and biomarker profiling. The study enrolled 10,198 participants. COPDGene has had 3 exams that include spirometry, diffusing capacity, lung CT scans and other measures; its current exam is ongoing. COPDGene examines the influence of age, sex, and race on the natural history of COPD, and the impact of comorbid conditions, chronic bronchitis, exacerbations, and asthma/COPD overlap.

Framingham Heart Study (FHS)[4]: FHS was initiated in 1948. Researchers recruited 5,209 men and women between the ages of 30 and 62 from the town of Framingham, Massachusetts, and began the first round of extensive physical examinations and lifestyle interviews that they would later analyze for common patterns related to CVD development. Since 1948, the subjects have returned to the study every two years for an examination consisting of a detailed medical history, physical examination, and laboratory tests, and in 1971, the study enrolled a second-generation cohort – 5,124 of the original participants' adult children and their spouses – to participate in similar examinations. The second examination of the Offspring cohort occurred eight years after the first examination, and subsequent examinations have occurred approximately every four years thereafter. In April 2002 the Study entered a new phase: the enrollment of a third generation of participants, the grandchildren of the original cohort. The first examination of the Third Generation Study was completed in July 2005 and involved 4,095 participants. Thus, the FHS has evolved into a prospective, community-based, three generation family study. In addition to research studies focused on risk factors, subclinical CVD and clinically apparent CVD, Framingham investigators have also collaborated with leading researchers from around the country and throughout the world on projects involving some of the major chronic illnesses in men and women, including dementia, osteoporosis and arthritis, nutritional deficiencies, eye diseases, hearing disorders, and chronic obstructive lung disease.

Hispanic Community Health Study/Study of Latinos (HCHS/SOL)[5-7]: HCHS/SOL is an ongoing population based prospective cohort study of 16,415 community dwelling Hispanic/Latino adults aged 18-74 years at baseline, recruited from four urban field centers with large populations of Hispanics/ Latinos (Bronx, NY; Chicago, IL; Miami, FL; and San Diego, CA). A two-stage area probability sample of households was selected, with stratification and over-sampling at each stage to ensure a diverse and representative sample.[6] Participants self-identified as Hispanic/Latino and of Cuban, Dominican, Mexican, Puerto Rican, Central American, South American, or other/more than one heritage. Study participants underwent an extensive clinic exam and assessments to determine baseline risk factors (2008-2011),[7] and annual telephone follow-up interviews for ascertainment of cardiovascular and pulmonary events. A second clinic visit was conducted in 2014-2017, and a third clinic visit is now in process (2020-2022). The overall retention rate as of December 2019 was 81.9%. The primary goals of the HCHS/SOL are to describe: (1) the prevalence and incidence of cardiovascular, pulmonary, and other major chronic conditions (2) the risk and/or protective factors associated with these conditions; and (3) the relationships between the initial sociodemographic and health profiles and future health events in the target population. The study to date has revealed a high prevalence of cardiovascular risk factors, with significant variability by Hispanic/Latino heritage and sociodemographic factors such as income and time in the United States.[5]

Jackson Heart Study (JHS)[8-10]: The JHS is a community-based cohort study evaluating risk factors for cardiovascular and related diseases among adult African Americans residing in the three counties (Hinds, Madison, and Rankin) that make up the Jackson, Mississippi metropolitan area. Data and biologic materials have been collected from 5,306 participants, including a nested family cohort of 1,498 members of 264 families. The age at enrollment for the unrelated cohort was 35-84 years; the family cohort included related individuals >21 years old. Participants have provided extensive medical and

© 2024 Oelsner EC et al. *JAMA Network Open.*

psychosocial histories and had an array of physical and biochemical measurements and diagnostic procedures during a baseline examination (2000-2004) and two follow-up examinations (2005-2008 and 2009-2012). Samples for genomic DNA were collected during the first two examinations. Annual follow-up interviews and cohort surveillance of cardiovascular events and mortality are continuing and a fourth examination is in progress.

Mediators of Atherosclerosis in South Asians Living in America (MASALA) study[11,12]: South Asians comprise almost one-quarter of the world's population and are the second fastest growing ethnic group in the US. The MASALA Study is a prospective cohort of South Asians called the MASALA study, which is closely tied to the Multi-Ethnic Study of Atherosclerosis (MESA), for valid cross-ethnic comparisons.[12] MASALA enrolled 906 South Asians in 2010-2013 and then added a new wave of 258 South Asian participants from 2017-2018, for a full cohort size of 1,164.[11] The original MASALA cohort has been followed for approximately 8.5 years, and completed a second clinical exam in early 2018. A third MASALA clinical exam is planned for 2022-2024. 75 papers have been published from MASALA to date, and the findings clearly show that the US South Asian population has a distinct phenotype compared to the other four race/ethnic groups studied in MESA. Major findings have included a higher prevalence of diabetes, ectopic adiposity and coronary artery calcium compared to MESA. The MASALA study findings have influenced guidelines for diabetes screening, lipid management, and raised awareness of South Asian CVD risk. MASALA is filling a large gap in scientific knowledge about CVD in a large, growing Asian American subgroup.

Multi-Ethnic Study of Atherosclerosis (MESA)[13]: MESA is a study of the characteristics of subclinical CVD (disease detected non-invasively before it has produced clinical signs and symptoms) and the risk factors that predict progression to clinically overt cardiovascular disease or progression of the subclinical disease. MESA researchers study a diverse, population-based sample of 6,814 men and women aged 45-84 without known clinical cardiovascular disease. Thirty-eight percent of the recruited participants are white, 28 percent African-American, 22 percent Hispanic, and 12 percent of Chinese descent. Participants were recruited from six field centers across the United States: Wake Forest University, Columbia University, Johns Hopkins University, University of Minnesota, Northwestern University and University of California – Los Angeles. At baseline, each participant received an extensive physical exam and determination of coronary artery calcification, ventricular mass and function, flow-mediated endothelial vasodilation, carotid intimal-medial wall thickness and presence of echogenic lucencies in the carotid artery, lower extremity vascular insufficiency, arterial wave forms, electrocardiographic (ECG) measures, standard coronary risk factors, sociodemographic factors, lifestyle factors, and psychosocial factors. Selected repetition of subclinical disease measures and risk factors at follow-up visits allows study of the progression of disease. Blood samples have been assayed for putative biochemical and genetic risk factors and stored for case-control studies. Participants are being followed for identification and characterization of cardiovascular disease events, including acute myocardial infarction and other forms of coronary heart disease (CHD), stroke, and heart failure; for CVD interventions; and for mortality. The first examination took place over two years, from July 2000 – July 2002. It has been followed by six examination periods that were 17-20 months in length. Participants have been contacted every 9 to 12 months throughout the study to assess clinical morbidity and mortality.

Northern Manhattan Study (NOMAS)[14]: NOMAS began in 1993 as a population-based incidence and case-control study. In 1998 (cycle 2) the study evolved into a prospective cohort study of 3,298 stroke-free, tri-ethnic, community subjects followed annually to detect stroke, MI, and death. Starting in 2003 (cycle 3), subclinical measures (brain MRI & carotid ultrasound) and the first complete

© 2024 Oelsner EC et al. *JAMA Network Open.*

neuropsychological (NP) battery were collected on 1290 members (MRI cohort). The project has remained productive through subsequent cycles. As the cohort aged, the specific aims grew to include not only vascular determinants of stroke but also cognitive decline, mild cognitive impairment (MCI) and dementia. NOMAS participates in collaborative studies on genetics, stroke, MRI markers, Alzheimer Disease and neurodegenerative diseases. One of the major interests of the study has been the exploration of inflammatory and infectious contributors to stroke risk, subclinical atherosclerotic and cerebrovascular disease, and cognitive decline. The NOMAS community cohort of 3,298 subjects was assembled from a population-based, random sample based on the following criteria: (1) resident of at least 3 months of Northern Manhattan; (2) randomly derived from a household with a telephone; (3) age 40 or older at baseline (changed to age 55 in 1998); and (4) no history of stroke. The 1,290 subjects in the MRI cohort (median age 70 at MRI; 60% women, 15% non-Hispanic White, 17% non-Hispanic Black, 66% Hispanic, 2% Other) were evaluated with a standardized brain MRI and NP battery between 2003-08. The cohort has been prospectively followed with annual telephone contacts, including the Telephone Interview for Cognitive Status (TICS), and 3 in depth neuropsychological evaluations at 5 year intervals in the MRI cohort. The aging cohort is representative of an elderly, urban, diverse community at risk for cognitive decline. A wealth of data was collected during baseline enrollment and at time of MRI and 1st NP visit, including socio-demographics, psychosocial and socioeconomic status (education, occupational attainment, insurance status), medical history, medications, risk factors, family history and other health data, behavioral/environmental factors, subclinical vascular measures, serum biomarkers (infectious burden, neuroimmune markers using a novel multiplex assay, HOMA index for insulin resistance, adiponectin, CRP, homocysteine), carotid imaging, echocardiographic imaging (LV, LA size), ambulatory BP and cardiac rhythm monitoring, brain MRI biomarkers (regional brain volumes, regional white matter lesion burden, hippocampal volumes, cortical thickness, covert infarcts, cerebral microbleeds, perivascular spaces, brain arterial diameters), and genetic markers (GWAS, ApoE4). Fasting blood was collected and stored at baseline and at MRI. Subjects had complete blood count, chemistry profile, total protein, albumin, calcium, markers of mineral metabolism (fibroblast growth factor 23, parathyroid hormone, 1,25OH and 25OH vitamin D, and phosphate), CRP, TNF receptor levels, IL-6, and serologies against some viral and bacterial pathogens. Fasting plasma levels were assayed for total and HDL cholesterol, lipoprotein (a), HDL particle size, triglycerides, lipoprotein-associated phospholipase A2, homocysteine, serum insulin levels, and adiponectin. Buffy coats and DNA were stored on 2433 subjects and ApoE4 genotype is available on the MRI cohort. We continue to follow the cohort with annual telephone contacts and a 4th NP assessment to track cognitive trajectories and adjudicate MCI and dementia. Cognitive, functional, quality of life, and social situation questions are assessed annually. The National Death Index is consulted periodically for those with unknown vital status. An I surveillance system at CUIMC detects hospitalizations, ED visits, and clinical visits. Remarkably, only 3 (0.38%) subjects are lost, and 11 (1.4%) have withdrawn from active participation.

Prevent Pulmonary Fibrosis (PrePF): PrePF has been investigating the clinical, physiologic and genetic phenotypes of interstitial lung disease (ILD) by focusing on families with two or more cases of ILD and individuals with sporadic IPF. It has recruited over 1200 families with two or more cases of pulmonary fibrosis. These families with pulmonary fibrosis include 2837 individuals with probable or definite idiopathic interstitial pneumonia (IIP) and 2404 unaffected FDRs. In addition, PrePF recruited over 10,000 individuals with sporadic idiopathic pulmonary fibrosis (IPF).

REasons for Geographic and Racial Differences in Stroke (REGARDS)[15]: the REGARDS cohort is one of the nation's largest, most comprehensive population-based cohorts, its innovative home- and telephone-based data collection is nimble and cost-efficient. REGARDS centrally recruited and initially examined 30,239 non-Hispanic Black and White men and women aged ≥45 years in 2003-7 by telephone

and in participant homes across the 48 contiguous US states (62% of US counties). Over 17 years, REGARDS has collected follow-up data by computer-assisted telephone interviews (CATI), participant collaboration in at-home tasks (i.e., actigraphy), and a 2nd in-home visit. REGARDS oversampled Black individuals and residents of the southeastern United States known as the Stroke Belt and 17% reside in rural areas. REGARDS currently follows ~11,000 surviving participants. Comprehensive available data include adjudicated health events, social determinants of health (SDOH), cognition, biomarkers and genomics. Participants currently have mean age 76.9 (range 57-105), are 37% Black, have high cardiovascular risk, and 54% reside in the southeast — all factors associated with COVID-19 risk and adverse outcomes. Participants are geocoded, and linked to administrative data such as EPA and Medicare. Biorepositories were assembled in 2003-2007 and 2013-2016.

Severe Asthma Research Program (SARP)[16]**:** SARP has been investigating the clinical, physiologic and molecular phenotypes of asthma since 2000. It is currently following ~400 deeply phenotyped asthma patients (60% severe), most with sputum samples, bronchoscopies, lung CTs, allergy status, spirometry and biobanking.

Subpopulations and Intermediate Outcome Measures in COPD Study (SPIROMICS)[17]: SPIROMICS is a multi-center, observational, longitudinal case-control study designed to guide future development of therapies for COPD by 1) providing robust criteria for sub-classifying COPD participants into groups most likely to benefit from a given therapy during a clinical trial, thereby improving the chances of successful outcome; and 2) identifying biomarkers and phenotypes that can be used as intermediate outcomes to reliably predict clinical benefit during therapeutic trials. The baseline exam included morphometric measures, spirometry, six-minute walk, an inspiratory and expiratory chest CT, and a set of standardized questionnaires. Biospecimens, including plasma, serum, DNA, urine and induced sputum, have been collected and stored. SPIROMICS has recruited 2,983 COPD cases and controls, 40-80 years old with 20+ or <1 pack-years of smoking at 12 US sites in 2010–2015.SPIROMICS has a baseline and 4 follow-up exams, that include spirometry, lung CT scans, sputum induction and, in a subset, bronchoscopies; its current exam is ongoing.

Strong Heart Study (SHS)[18,19]: SHS was designed to respond to the recommendations from the Subcommittee on Cardiovascular and Cerebrovascular Disease of the Secretary of Health and Human Service's Task Force on Black and Minority Health that concluded that information on cardiovascular disease (CVD) in American Indians was inadequate. In its initial stages, the SHS included three components. The first was a survey to determine cardiovascular disease mortality rates from 1984 to 1994 among tribal members aged 35-74 years of age residing in the 3 study areas (the community mortality study). The second was the clinical examination of 4,500 eligible tribal members. The third component is the morbidity and mortality (M&M) surveillance of these 4,500 participants. SHS has completed three clinical examinations of the original Cohort in Phase I 1989-1991; Phase II: 1993-1995; 1998-1999, respectively. In Phases III-V, SHS expanded to include genetic epidemiologic studies and family-based genetics studies due to the importance of genetics in the occurrence of CVD. Phase VI was a surveillance of the original SHS cohort and of the SHS family study participants to better understand CVD, cancer, liver disease, and inflammation in American Indians. Phase VII is currently underway with continued surveillance beginning February 2019 for a seven-year duration. The SHS Phase VII exam serves as a platform for in-depth ancillary studies that are funded outside of the SHS contracts.

Follow-up for SARS-CoV-2 infection in C4R

© 2024 Oelsner EC et al. *JAMA Network Open.*

Of C4R questionnaire respondents, follow-up for incident infection was complete for 41,461 (84.0%) over the first six months; for 35,790 (72.5%) over the first 12 months; and 10,795 (21.9%) over the first 24 months. Details regarding timing of questionnaire administration are provided in **eTable 2**. The flow chart for sample selection is provided as **eFigure 1**.

Covariate definition

Age, sex, and educational attainment were self-reported at enrollment into each cohort study. Age at enrollment into C4R in March 2020 was calculated from age at cohort enrollment. Since categories to self-report race and ethnicity differed by cohort, they were harmonized into a single classification (American Indian, Asian, Non-Hispanic Black, Hispanic, Non-Hispanic White).

Pre-pandemic health measures were obtained for each individual cohort from the examination closest to the time of C4R enrollment, with a median interval of 5 years (25th percentile, 4; 75th percentile, 12) (**eTable 1**).[20] Height, weight, and systolic and diastolic blood pressure were measured using standardized methods. Blood glucose and creatinine were measured in fasting blood samples. BMI was categorized by current CDC thresholds. Current smoking status was assessed at the most recent cohort examination by self-report, with biochemical verification in a subset. Medication use was self-reported or assessed via medication inventories. Diabetes was self-reported or defined by fasting blood glucose (≥126 mg/dL) or use of hypoglycemic medications. Hypertension was defined by blood pressure (≥140/90 mmHg) or use of anti-hypertensive medications. Estimated glomerular filtration rate (eGFR) was calculated by the Chronic Kidney Disease Epidemiology Collaboration equation. Chronic Kidney Disease (CKD) was defined by eGFR < 45 ml/min/1.73m^2.[21] A history of clinical cardiovascular disease (CVD) was defined as a prior self-report of myocardial infarction, coronary heart disease, or heart failure, or the occurrence of relevant, adjudicated health events over cohort follow-up prior to the pandemic. A history of asthma, COPD, emphysema, and chronic bronchitis was self-reported. A history of elevated depressive symptoms was defined by a score of 10 or greater on the 10-item Centers for Epidemiologic Depression (CES-D) scale.

Analyses

For Cox proportional hazards models, the proportional hazards assumption was confirmed by residual plots. Participants who had not recovered by 90 days were censored at 90 days on the conservative assumption that proportionality may not hold among outliers with recovery times significantly greater than 90 days. For mediation analyses, time-to-recovery was modeled using a Weibull accelerated failure time model, and ordinal logistic regression was used to model infection severity.

Multiple imputation

Multiple imputation of missing data among participants with a history of infection was performed based on the variables in the main model as well as infection severity, region, source cohort, recovery status, and observed recovery or censoring time transformed using the Nelson-Aalen estimate.[22] We assumed that data were missing at random. The imputation was conducted using the "mice" package in the R statistical software.[23] Participants with missing recovery time were excluded from the primary analyses. Results across 10 imputed datasets were combined by Rubin's Rule.

© 2024 Oelsner EC et al. *JAMA Network Open.*

eTable 1. Characteristics of Participants in C4R Cohorts, United States, March 1, 2020.

Cohort	N	Original enrollment	Most recent pre-pandemic exam [a]	Current age range, years	Sex, % Women	Race and ethnicity, % NHW	B	H/L	As	Am Ind	Other	Original research focus
ARIC	6,690	1987-89	Visit 7 (2018-19)	75-97	63	77	23	0[b]	0	0	0	Cardiovascular
CARDIA	4,590	1985-86	Year 30 (2015-16)	53-66	56	50	50	0	0	0	0	Cardiovascular
COPDGene	7,731	2007-12	Phase 3 (2018-22)	50-90	48	65	35	0	0	0	0	Pulmonary
FHS	7,339	1971-2005	Offspring Exam 9 (2011-14)<br>Gen3 Exam 3 (2016-19)	26-108	56	86	3	4	0	0	7	Cardiovascular
HCHS/SOL	13,142	2008-11	Exam 2 (2014-17)	30-87	60	0	0	100	0	0	0	Cardiovascular
JHS	2,444	2000-04	Exam 3 (2009-13)	38-102	63	0	100	0	0	0	0	Cardiovascular
MASALA	1,132	2010-13	Exam 2 (2016-18)	50-94	47	0	0	0	100	0	0	Cardiovascular
MESA	4,683	2000-02	Exam 6 (2016-18)	65-103	56	38	27	24	12	0	0	Cardiovascular
NOMAS	1,256	1993-2003	Visit 3 (2016-21)	62-106	65	12	14	72	0	1	0	Neurologic
PrePF	5,000	2000-13	Exam 1 (2015-16)	40-80	55	92	3	3	0	0	0	Pulmonary
REGARDS	12,766	2003-07	Exam 2 (2013-16)	57-105	58	62	38	0	0	0	0	Neurologic
SARP	397	2000-present	Visit 8 (2019)	18-80	65	75	25	0	0	0	0	Pulmonary
SPIROMICS	2,273	2010-15	Visit 5 (2018-21)	47-87	48	82	4	4	0	0	0	Pulmonary
SHS	2,915	1984-94	Phase VI (2013-15)	31-105	62	0	0	0	0	100	0	Cardiovascular

Am Ind = American Indian; As = Asian American; B = Black; H/L = Hispanic/Latinx; NHW = Non-Hispanic White.

ARIC = Atherosclerosis Risk in Communities Study; C4R = Collaborative Cohort of Cohorts for COVID-19 Research; CARDIA = Coronary Artery Risk Development in Young Adults; COPDGene= Genetic Epidemiology of COPD; FHS = Framingham Heart Study; HCHS/SOL = Hispanic Community Health Study/Study of Latinos; JHS = Jackson Heart Study; MASALA = Mediators of Atherosclerosis in South Asians Living in America; MESA = Multi-Ethnic Study of Atherosclerosis; NOMAS = Northern Manhattan Study; PrePF = Prevent Pulmonary Fibrosis; REGARDS = Reasons for Geographic and Racial Differences in Stroke; SARP = Severe Asthma Research Program; SPIROMICS = Subpopulations and Intermediate Outcome Measures in COPD Study; SHS = Strong Heart Study

[a] For C4R participants who did not attend the most recent pre-pandemic cohort exam, the most recent measure from any prior cohort exam was used.

[b] ARIC did not inquire regarding Hispanic/Latino ethnicity, hence White participants cannot be definitely defined as non-Hispanic.

© 2024 Oelsner EC et al. *JAMA Network Open.*

eTable 2. Questionnaire Administration, Including Response Rates and Availability of Complete Data for Analysis, by COHORT.

Cohort	Enrollment estimate for C4R*	Responded to questionnaire, of enrollment estimate*, No. (%)	Infection self reported, of respondents, No. (%)	Valid data on recovery by 90 days, of infections, No. (%)	Average date of questionnaire completion (25th and 75th percentiles)
ARIC	5046	5504 (109.1)	450 (8.2)	257 (57.1)	July 25, 2021 (January 25, 2021; November 30, 2021)
CARDIA	4221	2815 (66.6)	429 (15.2)	311 (72.5)	July 31, 2021 (March 14, 2021; November 2, 2021)
COPDGene	4000	4164 (104.1)	432 (10.4)	317 (73.4)	May 31, 2021 (June 30, 2020; February 4, 2022)
FHS	7339	3206 (43.7)	514 (16.0)	447 (87.0)	July 23, 2021 (March 10, 2021; December 16, 2021)
HCHS/SOL	8400	11304 (134.6)	2296 (20.3)	1901 (82.8)	September 2, 2021 (January 17, 2021; March 24, 2022)
JHS	2317	2314 (99.9)	249 (10.8)	185 (74.3)	August 2, 2021 (March 8, 2021; January 15, 2022)
MASALA	500	571 (114.2)	38 (6.7)	31 (81.6)	July 5, 2021 (February 21, 2021; October 7, 2021)
MESA	4683	3478 (74.3)	292 (8.4)	213 (72.9)	July 30, 2021 (August 20, 2021; December 10, 2021)
NOMAS	1256	849 (67.6)	169 (19.9)	130 (76.9)	July 27, 2021 (May 15, 2021; August 2, 2021)
PrePF	2500	628 (25.1)	115 (18.3)	90 (78.3)	July 12, 2021 (March 31, 2021; October 14, 2021)
REGARDS	8000	10268 (128.4)	1169 (11.4)	337 (28.8)	August 29, 2021 (July 19, 2021; September 14, 2021)
SARP	380	387 (101.8)	62 (16.0)	42 (67.7)	July 28, 2021 (March 30, 2021; January 18, 2022)
SHS	2701	1967 (72.8)	630 (32.0)	361 (57.3)	November 14, 2021 (August 23, 2021; February 23, 2022)
SPIROMICS	1800	1539 (85.5)	146 (9.5)	86 (58.9)	May 21, 2021 (October 29, 2020; December 9, 2021)

*Estimates of the number of participants who would were developed by each cohort prior to study enrollment. In several cases, a greater number of participants was enrolled than was originally estimated.

Row percents reported. ARIC = Atherosclerosis Risk in Communities Study; C4R = Collaborative Cohort of Cohorts for COVID-19 Research; CARDIA = Coronary Artery Risk Development in Young Adults; COPDGene= Genetic Epidemiology of COPD; FHS = Framingham Heart Study; HCHS/SOL = Hispanic Community Health Study/Study of Latinos; JHS = Jackson Heart Study; MASALA = Mediators of Atherosclerosis in South Asians Living in America; MESA = Multi-Ethnic Study of Atherosclerosis; NOMAS = Northern Manhattan Study; PrePF = Prevent Pulmonary Fibrosis; REGARDS = Reasons for Geographic and Racial Differences in Stroke; SARP = Severe Asthma Research Program; SPIROMICS = Subpopulations and Intermediate Outcome Measures in COPD Study; SHS = Strong Heart Study

© 2024 Oelsner EC et al. *JAMA Network Open.*

eTable 3. Definition of Infection, Infection Severity, and Time to Recovery Via C4R Wave 1 Questionnaires, Administered April 1, 2020, Through May 29, 2022.

Question	N, Cohorts
Do you think that you have had COVID-19?	14
Did a healthcare provider ever tell you that you had COVID-19?	13 [a]
Have you ever had a test that showed you had COVID-19?	14
When you knew or thought that you had COVID-19 the first time, did you have any symptoms?	14
Since March 1, 2020, have you had an overnight stay in a hospital for any illness related to COVID-19?	14
While in the hospital, did you have any of the following treatments: A breathing tube or ventilator? ICU monitoring?	10 [b]
Following your COVID-19 infection, would you say that you are completely recovered from COVID-19 now?	13[a]
How many days did it take you to recover from COVID-19?	11[c]
Have you received a vaccine for COVID-19?	4[d]
When were you vaccinated?	4[d]
How many doses did you receive?	4[d]

[a]Not included in Wave 1 Questionnaire in REGARDS.

[b]Not included in Wave 1 Questionnaire in ARIC, CARDIA, HCHS/SOL, or REGARDS.

[c]Not included in Wave 1 Questionnaire in COPDGene, REGARDS or SHS.

[d]Included in cohorts that initiated the Wave 1 Questionnaire in 2021.

© 2024 Oelsner EC et al. *JAMA Network Open.*

eTable 4. Definition of Infection, Infection Severity, and Time to Recovery Via C4R Wave 2 Questionnaires, Administered March 1, 2021, Through February 28, 2023.

Domain	Question	N, Cohorts
COVID-19 TESTING	Have you ever had any kind of test for COVID-19?	14
	Have you ever had a test that showed you had COVID-19? Please include all types of tests.	14
	When was it that you first had a test that showed you had COVID-19?	14
COVID-19 SELF-REPORT	Do you think that you have had COVID-19?	14
	When do you think you FIRST had COVID-19?	14
	Were you tested at that time?	11
COVID-19 HOSPITALIZATION	Since March 2020, have you had an overnight stay in a hospital for any illness related to COVID-19?	14
COVID-19 SYMPTOMS	When you knew or thought that you had COVID-19, did you have any symptoms?	14
COVID-19 RECOVERY	Following your COVID-19 infection, would you say that you are completely recovered from COVID-19 now?	14
COVID-19 VACCINATION	Have you received a vaccine for COVID-19?	14
	When were you vaccinated?	14
	How many doses did you receive?	14

© 2024 Oelsner EC et al. *JAMA Network Open.*

eTable 5. Classification of Selected COVID-19 Outcomes Available in C4R as Confirmed vs Probably, and Number (Percent) of Cases in the Analytic Sample (n = 4708).

Outcome	Confirmed cases		Probable cases	
	**Criteria for confirmed case (any)**	No. (%)	**Criteria for probable case (any)**	No. (%)
**Infection**	Adjudicated definite COVID-19 infection (N=323)<br>Self-/proxy-report of positive test for SARS-CoV-2 (N=3730)<br>Anti-nucleocapsid protein IgG on C4R serology(N=434)<br>Anti-spike protein IgG on C4R serology without COVID vaccination (N=143)	3825 (81.2)	Adjudicated probable COVID-19 infection (N=4)<br>Self-/proxy-report of self- or healthcare provider-diagnosed COVID-19 without confirmatory testing (N=883)	883 (18.8)
**Recovery to usual state of health**	Self-report of recovery to usual state of health in the context of definite infection	2997 (63.7)	Self-report of recovery to usual state of health in the context of probable infection	659 (14.0)
**Hospitalization**	Adjudicated hospitalization definitely caused by COVID-19	292 (6.2)	Adjudicated hospitalization probably caused by COVID-19 (N=11)<br>Self-/proxy-report of hospitalization for COVID-19 (N=294)	305 (6.5)

Percentages based on main analysis sample (N=4,708). Numbers indicated for specific criteria may not add to the totals since they are not mutually exclusive.

© 2024 Oelsner EC et al. *JAMA Network Open.*

eTable 6. Missingness of Covariate Data Among Participants Included in the Analysis.

Covariate	Participants with missing data, No. (% of analysis sample)
Age	49 (1.0%)
Sex	2 (0.0%)
Race and ethnicity	10 (0.2%)
Educational attainment	133 (2.8%)
Smoking status	28 (0.6%)
BMI	231 (4.9%)
Diabetes	165 (3.5%)
Hypertension	148 (3.1%)
Cardiovascular disease	766 (16.3%)
eGFR	336 (7.1%)
COPD	780 (16.6%)
Asthma	868 (18.4%)
Depressive symptoms	1697 (36.0%)
Region	103 (2.2%)
Acute infection severity	0 (0.0%)
Vaccination status	741 (15.7%)
Infection wave	509 (10.8%)
Source cohort	0 (0.0%)
Time-to-recovery	0 (0.0%)
Recovery status	0 (0.0%)

BMI = body mass index. COPD = chronic obstructive pulmonary disease. eGFR = estimated glomerular filtration rate.

© 2024 Oelsner EC et al. *JAMA Network Open.*

eTable 7. Comparison of Characteristics of Infected C4R Participants Included vs Not Included in the Analytic Sample.[a]

Correlate	Categories	Not in analysis sample due to missing recovery data, No. (%)	Analysis sample, No. (%)
Total		3,127 (100)	4,708 (100)
Age	< 50 years	297 (9.7)	932 (20.0)
	50 – 64 years	848 (27.6)	2020 (43.4)
	65 – 79 years	1320 (42.9)	1330 (28.5)
	80+ years	611 (19.9)	377 (8.1)
Sex	Men	1274 (40.8)	1755 (37.3)
	Women	1852 (59.2)	2951 (62.7)
Race and ethnicity	American Indian	410 (13.1)	371 (7.9)
	Asian	29 (0.9)	50 (1.1)
	Black, Non-Hispanic	809 (25.9)	622 (13.2)
	Hispanic	540 (17.3)	2086 (44.3)
	White, Non-Hispanic	1332 (42.6)	1569 (33.3)
Educational attainment	< High School	384 (12.5)	683 (14.9)
	High School	869 (28.2)	1139 (24.9)
	Some College	689 (22.4)	846 (18.5)
	College degree	1139 (37.0)	1907 (41.7)
Smoking Status	Never	1465 (47.0)	2599 (55.5)
	Former	1148 (36.9)	1452 (31.0)
	Current	501 (16.1)	629 (13.4)
BMI	< 25 kg/m^2	600 (19.6)	904 (20.2)
	25-29 kg/m^2	1057 (34.5)	1633 (36.5)
	30+ kg/m^2	1405 (45.9)	1940 (43.3)
Diabetes	Absent	2312 (75.1)	3696 (81.4)
	Present	765 (24.9)	847 (18.6)
Hypertension	Absent	1387 (44.9)	2786 (61.1)
	Present	1704 (55.1)	1774 (38.9)
Cardiovascular disease	Absent	1770 (90.4)	3707 (94.0)
	Present	188 (9.6)	235 (6.0)
eGFR < 45	Absent	2779 (97.4)	4234 (96.8)
	Present	75 (2.6)	138 (3.2)
Asthma	Absent	1648 (85.6)	3250 (84.6)
	Present	277 (14.4)	590 (15.4)
COPD, emphysema, chronic bronchitis	Absent	1782 (90.3)	3612 (92.0)
	Present	192 (9.7)	590 (15.4)
Depressive symptoms	Absent	1492 (85.1)	2528 (84.0)
	Present	261 (14.9)	482 (16.0)
Vaccination prior to infection	No	1178 (66.2)	3214 (81.9)
	Yes	602 (33.8)	709 (18.1)
Infection wave	First (Wild type)	262 (12.9)	938 (22.3)
	Second (Wild type)	224 (11.0)	543 (12.9)
	Third (Alpha)	610 (30.0)	1425 (33.9)
	Fourth (Alpha)	208 (10.2)	251 (6.0)
	Fifth (Delta)	413 (20.3)	508 (12.1)
	Sixth (Omicron)	318 (15.6)	534 (12.7)

© 2024 Oelsner EC et al. *JAMA Network Open.*

Correlate	Categories	Not in analysis sample due to missing recovery data, No. (%)	Analysis sample, No. (%)
COVID severity	Asymptomatic	362 (11.6)	0 (0.0)
	Symptomatic, non-hospitalized	893 (28.6)	3513 (74.6)
	Symptoms unknown, non-hospitalized	1358 (43.5)	598 (12.7)
	Hospitalized, non-critical	281 (9.0)	449 (9.5)
	Hospitalized, critical	53 (1.7)	148 (3.1)
	Fatal	174 (5.6)	0 (0.0)
Study	ARIC	365 (11.7)	257 (5.5)
	CARDIA	159 (5.1)	311 (6.6)
	COPDGene	213 (6.8)	317 (6.7)
	FHS	102 (3.3)	447 (9.5)
	HCHS/SOL	463 (14.8)	1901 (40.4)
	JHS	85 (2.7)	185 (3.9)
	MASALA	12 (0.4)	31 (0.7)
	MESA	177 (5.7)	213 (4.5)
	NOMAS	41 (1.3)	130 (2.8)
	PrePF	30 (1.0)	90 (1.9)
	REGARDS	972 (31.1)	337 (7.2)
	SARP	29 (0.9)	42 (0.9)
	SHS	406 (13.0)	361 (7.7)
	SPIROMICS	73 (2.3)	86 (1.8)

[a]No imputed data included.

© 2024 Oelsner EC et al. *JAMA Network Open.*

eTable 8. Correlates of Recovery by 90 Days After SARS-CoV-2 Infection After Multivariable Adjustment, Adjusted for Disease Severity.

Correlate	Categories	Hazard ratio for recovery[a] (95% CI)	p-value
Age (ref: <50 years)	50 – 64 years	1.06 (0.96-1.17)	0.24
	65 – 79 years	1.11 (0.98-1.25)	0.10
	80+ years	1.13 (0.93-1.36)	0.21
Sex (ref: men)	Women	0.83 (0.77-0.89)	<0.001
Educational attainment (ref: College degree)	< High School	1.06 (0.95-1.18)	0.32
	High School	1.05 (0.96-1.14)	0.33
	Some College	1.02 (0.93-1.13)	0.67
Smoking Status (ref: Never)	Former	0.95 (0.88-1.03)	0.25
	Current	0.92 (0.83-1.03)	0.15
BMI (ref: <25 kg/m^2)	25-29 kg/m^2	1.02 (0.93-1.13)	0.66
	30+ kg/m^2	0.93 (0.84-1.02)	0.14
Diabetes (ref: absent)	Present	0.98 (0.89-1.08)	0.68
Hypertension (ref: Absent)	Present	0.97 (0.89-1.05)	0.39
Cardiovascular disease (ref: absent)	Present	0.86 (0.73-1.01)	0.067
eGFR < 45 $ml/min/1.73m^2$ (ref: eGFR ≥45 $ml/min/1.73m^2$)	Present	0.87 (0.71-1.07)	0.18
Asthma (ref: absent)	Present	1.00 (0.90-1.10)	0.92
COPD (ref: absent)	Present	0.90 (0.77-1.06)	0.20
Elevated depressive symptoms (ref: absent)	Present	0.94 (0.84-1.05)	0.25
Vaccination prior to infection (ref: no)	Yes	1.22 (1.05-1.43)	0.014
Infection wave (ref: 1st, WT, Spring 2020)	Second (WT, Summer/Fall 2020)	1.07 (0.95-1.21)	0.26
	Third (Alpha, Winter 2020-21)	0.98 (0.89-1.09)	0.75
	Fourth (Spring 2021)	0.92 (0.78-1.08)	0.31
	Fifth (Delta, Summer 2021)	1.00 (0.85-1.18)	0.99
	Sixth (Omicron, Winter 2021-22)	1.25 (1.05-1.48)	0.011
Acute infection severity (ref: outpatient	Non-critical hospitalization	0.59 (0.52-0.67)	<0.001
	Critical hospitalization	0.46 (0.36-0.57)	<0.001

Ref = referent. BMI = Body Mass Index. CI = confidence interval. WT = Wild Type.

[a]Cox proportional hazards models were estimated to assess associations of time-to-recovery with the correlates of interest. Estimates were generated from models adjusted for all the correlates listed in the table. Hazards ratios (HRs) greater than one indicate faster recovery, whereas HRs less than one indicate slower recovery.

© 2024 Oelsner EC et al. *JAMA Network Open.*

eTable 9. Restricted Mean Recovery Time From Reinfections (n = 212) in Strata of Covariates Identified in the Main Model.[a]

Correlate	Categories	Restricted mean recovery time, days[b] (95% CI)	p-value for log-rank test
Overall		38.27 (33.53-43.00)	
Sex	Women	42.31 (36.12-48.50)	0.026
	Men	31.48 (24.47-38.49)	
Cardiovascular disease	Absent	38.51 (33.47-43.55)	0.86
	Present	36.64 (17.46-55.83)	
Vaccination prior to infection	Yes	35.35 (26.22-44.47)	0.48
	No	37.56 (31.80-43.31)	
Infection wave	Omicron	28.59 (16.90-40.29)	0.09
	Pre-Omicron	39.82 (34.69-44.94)	

[a]Restricted mean recovery time estimated from Kaplan Meier curves censored at 90 days following re-infection. Log rank tests were used to test for differences in restricted mean recovery time according covariates.

© 2024 Oelsner EC et al. *JAMA Network Open.*

eTable 10. Main Correlates of Recovery in Time to Event Models, Including Cases of Asymptomatic or Fatal SARS-CoV-2 Infection.

Correlate	Categories	Hazard ratio[a] (95% CI) for recovery, including asymptomatic cases (N = 4,959)	Hazard ratio[a] (95% CI) for recovery, including fatal cases[b] (N=4,974)
Age (ref: <50 years)	50 – 64 years	1.04 (0.94, 1.14)	1.02 (0.93, 1.13)
	65 – 79 years	1.06 (0.94, 1.19)	0.96 (0.85, 1.08)
	80+ years	1.07 (0.90, 1.28)	0.68 (0.57, 0.82)
Sex (ref: men)	Women	0.86 (0.80, 0.92)	0.87 (0.81, 0.94)
Educational attainment (ref: College)	< High School	1.04 (0.94, 1.16)	1.00 (0.90, 1.11)
	High School	1.05 (0.96, 1.15)	1.01 (0.93, 1.11)
	Some College	1.02 (0.93, 1.12)	1.02 (0.93, 1.13)
Smoking Status (ref: Never)	Former	0.96 (0.88, 1.03)	0.93 (0.85, 1.01)
	Current	0.91 (0.82, 1.01)	0.86 (0.77, 0.97)
BMI (ref: <25 kg/m^2)	25-29 kg/m^2	1.03 (0.94, 1.13)	1.02 (0.93, 1.13)
	30+ kg/m^2	0.92 (0.84, 1.01)	0.93 (0.84, 1.02)
Diabetes (ref: absent)	Present	0.93 (0.85, 1.02)	0.90 (0.82, 0.99)
Hypertension (ref: Absent)	Present	0.94 (0.87, 1.02)	0.92 (0.84, 1.00)
Cardiovascular disease (ref: absent)	Present	0.85 (0.73, 1.00)	0.84 (0.72, 0.97)
eGFR < 45 ml/min/1.73m^2 (ref: eGFR ≥45 ml/min/1.73m^2)	Present	0.88 (0.72, 1.08)	0.88 (0.71, 1.09)
Asthma (ref: absent)	Present	0.98 (0.89, 1.08)	1.00 (0.89, 1.13)
COPD (ref: absent)	Present	0.86 (0.74, 1.00)	0.89 (0.75, 1.06)
Elevated depressive symptoms (ref: absent)	Present	0.91 (0.81, 1.02)	0.93 (0.82, 1.04)
Vaccination prior to infection (ref: no)	Yes	1.32 (1.14, 1.52)	1.31 (1.08, 1.60)
Infection wave (ref: 1st, WT, Spring 2020)	Second (WT, Summer/Fall 2020)	1.09 (0.97, 1.23)	1.04 (0.92, 1.18)
	Third (Alpha, Winter 2020-21)	1.01 (0.92, 1.12)	1.00 (0.91, 1.11)
	Fourth (Spring 2021)	1.01 (0.86, 1.18)	0.95 (0.81, 1.12)
	Fifth (Delta, Summer 2021)	0.99 (0.84, 1.15)	1.02 (0.85, 1.23)
	Sixth (Omicron, Winter 2021 – present)	1.25 (1.06, 1.48)	1.35 (1.10, 1.65)

[a]Cox proportional hazards models were performed to assess associations of time-to-recovery with the correlates of interest. Estimates were generated from models adjusted for all of the covariates listed in the table. Hazards ratios (HRs) greater than one indicate faster recovery, whereas HRs less than one indicate slower recovery. Estimates with a p-value of <0.05 are indicated in bold text.

[b]Fatal cases include participants who reported recovery information but died within 90 days (N=15, shown in Supplementary Figure 1) plus participants with fatal infections who did not report recovery time (N=236).

© 2024 Oelsner EC et al. *JAMA Network Open.*

eTable 11. Main Correlates of Recovery in Time to Event Models, After Exclusion of Probable (Nondefinite) Cases.[a]

Correlate	Categories	Hazard ratio for recovery[b] (95% CI)	p-value
Age (ref: <50 years)	50 – 64 years	1.05 (0.94-1.17)	0.40
	65 – 79 years	1.09 (0.95-1.24)	0.21
	80+ years	1.08 (0.88-1.32)	0.46
Sex (ref: men)	Women	0.85 (0.79-0.92)	<0.001
Educational attainment (ref: College)	< High School	1.03 (0.91-1.16)	0.64
	High School	1.06 (0.96-1.17)	0.28
	Some College	0.99 (0.89-1.10)	0.83
Smoking Status (ref: Never)	Former	0.95 (0.87-1.04)	0.31
	Current	0.88 (0.77-0.99)	0.035
BMI (ref: <25 kg/m^2)	25-29 kg/m^2	0.97 (0.87-1.08)	0.57
	30+ kg/m^2	0.89 (0.80-0.99)	0.028
Diabetes (ref: absent)	Present	0.92 (0.82-1.02)	0.098
Hypertension (ref: Absent)	Present	0.95 (0.87-1.03)	0.22
Cardiovascular disease (ref: absent)	Present	0.82 (0.68-1.00)	0.049
eGFR < 45 ml/min/1.73m^2 (ref: eGFR ≥45 ml/min/1.73m^2)	Present	0.93 (0.74-1.18)	0.56
Asthma (ref: absent)	Present	0.97 (0.87-1.09)	0.64
COPD (ref: absent)	Present	0.93 (0.78-1.10)	0.39
Elevated depressive symptoms (ref: absent)	Present	0.89 (0.79-1.01)	0.076
Vaccination prior to infection (ref: no)	Yes	1.30 (1.12-1.52)	0.001
Infection wave (ref: 1st, WT, Spring 2020)	Second (WT, Summer/Fall 2020)	1.03 (0.90-1.18)	0.63
	Third (Alpha, Winter 2020-21)	0.92 (0.83-1.03)	0.16
	Fourth (Spring 2021)	0.92 (0.77-1.10)	0.34
	Fifth (Delta, Summer 2021)	0.92 (0.78-1.09)	0.34
	Sixth (Omicron, Winter 2021-present)	1.20 (1.00-1.45)	0.047

[a]Excludes 862 probable (non-definite) infections for a sample size of 3659.

[b]Cox proportional hazards models were performed to assess associations of time-to-recovery with the correlates of interest. Estimates were generated from models adjusted for all of the covariates listed in the table. Hazards ratios (HRs) greater than one indicate faster recovery, whereas HRs less than one indicate slower recovery.

© 2024 Oelsner EC et al. *JAMA Network Open.*

eTable 12. Main Correlates of Recovery in Time to Event Models, Without Stratification by Cohort, Adjusting for Race, Ethnicity, and Cohort.

Correlate	Categories	Hazard ratio (95% CI) for recovery<br>No stratification by cohort	Hazard ratio (95% CI) for recovery<br>No stratification by cohort, adjusted for cohort	Hazard ratio (95% CI) for recovery<br>No stratification by cohort, adjusted for race and ethnicity
Age (ref: <50 years)	50 – 64 years	1.10 (1.00, 1.21)	1.04 (0.95, 1.15)	1.06 (0.96, 1.16)
	65 – 79 years	1.08 (0.97, 1.20)	1.03 (0.92, 1.16)	1.03 (0.92, 1.15)
	80+ years	1.11 (0.95, 1.30)	1.00 (0.83, 1.21)	1.06 (0.91, 1.24)
Sex (ref: men)	Women	0.86 (0.80, 0.93)	0.86 (0.80, 0.92)	0.86 (0.80, 0.92)
Educational attainment (ref: College)	< High School	1.06 (0.96, 1.18)	1.04 (0.94, 1.16)	1.05 (0.95, 1.17)
	High School	1.00 (0.92, 1.09)	1.04 (0.95, 1.14)	1.02 (0.93, 1.11)
	Some College	0.99 (0.90, 1.09)	1.01 (0.92, 1.12)	1.00 (0.91, 1.10)
Smoking Status (ref: Never)	Former	0.88 (0.81, 0.95)	0.95 (0.88, 1.03)	0.89 (0.82, 0.96)
	Current	0.79 (0.71, 0.88)	0.93 (0.82, 1.03)	0.82 (0.74, 0.92)
BMI (ref: <25 kg/m^2)	25-<30 kg/m^2	1.01 (0.92, 1.11)	1.01 (0.92, 1.11)	1.01 (0.92, 1.12)
	30+ kg/m^2	0.91 (0.83, 1.00)	0.91 (0.83, 1.00)	0.91 (0.83, 1.01)
Diabetes (ref: absent)	Present	0.92 (0.83, 1.01)	0.94 (0.85, 1.03)	0.94 (0.85, 1.03)
Hypertension (ref: Absent)	Present	0.92 (0.85, 1.00)	0.96 (0.88, 1.04)	0.93 (0.86, 1.01)
Cardiovascular disease (ref: absent)	Present	0.87 (0.73, 1.02)	0.84 (0.71, 0.99)	0.84 (0.72, 0.99)
eGFR < 45 ml/min/1.73m^2 (ref: eGFR ≥45 ml/min/1.73m^2)	Present	0.97 (0.80, 1.19)	0.91 (0.74, 1.12)	0.95 (0.78, 1.17)
Asthma (ref: absent)	Present	1.03 (0.93, 1.14)	1.00 (0.90, 1.10)	1.03 (0.94, 1.14)
COPD (ref: absent)	Present	0.72 (0.62, 0.84)	0.87 (0.74, 1.02)	0.71 (0.61, 0.83)
Elevated depressive symptoms (ref: absent)	Present	0.81 (0.72, 0.90)	0.92 (0.82, 1.03)	0.91 (0.82, 1.02)
Vaccination status at time of infection (ref: unvaccinated)	Vaccinated	1.32 (1.13, 1.54)	1.30 (1.12, 1.52)	1.32 (1.13, 1.54)
Infection wave (ref: First, WT, Spring 2020)	Second (WT, Summer/Fall 2020)	1.13 (1.00, 1.27)	1.06 (0.94, 1.20)	1.13 (1.01, 1.28)
	Third (Alpha, Winter 2020-21)	1.05 (0.95, 1.15)	0.99 (0.90, 1.10)	1.08 (0.98, 1.19)
	Fourth (Spring 2021)	1.02 (0.87, 1.20)	0.94 (0.80, 1.11)	1.02 (0.87, 1.20)
	Fifth (Delta, Summer 2021)	1.06 (0.90, 1.24)	0.99 (0.85, 1.17)	1.07 (0.91, 1.25)
	Sixth (Omicron, Winter 2021-present)	1.28 (1.08, 1.53)	1.26 (1.06, 1.49)	1.30 (1.09, 1.54)

© 2024 Oelsner EC et al. *JAMA Network Open.*

Correlate	Categories	Hazard ratio (95% CI) for recovery<br>No stratification by cohort	Hazard ratio (95% CI) for recovery<br>No stratification by cohort, adjusted for cohort	Hazard ratio (95% CI) for recovery<br>No stratification by cohort, adjusted for race and ethnicity
Race and ethnicity (ref: non-Hispanic white)	AIAN			0.64 (0.53, 0.78)
	Asian			1.17 (0.84, 1.63)
	Non-Hispanic Black			1.11 (0.99, 1.24)
	Hispanic/Latino			1.00 (0.92, 1.10)
Study (ref: ARIC)	CARDIA		1.12 (0.90, 1.40)	
	COPDGene		0.32 (0.25, 0.41)	
	FHS		0.93 (0.76, 1.14)	
	HCHS/SOL		0.86 (0.71, 1.03)	
	JHS		1.03 (0.82, 1.30)	
	MASALA		1.04 (0.66, 1.67)	
	MESA		0.87 (0.70, 1.08)	
	NOMAS		0.93 (0.72, 1.21)	
	PrePF		1.22 (0.91, 1.63)	
	REGARDS		1.00 (0.82, 1.03)	
	SARP		0.89 (0.59, 1.33)	
	SHS		0.53 (0.41, 0.68)	
	SPIROMICS		1.10 (0.82, 1.47)	

© 2024 Oelsner EC et al. *JAMA Network Open.*

eTable 13. Main Correlates of Recovery in Time to Event Models, After Exclusion of Selected Cohorts.

N = 3860 (3027 recovered by 90 days)	Categories	Hazard ratio (95% CI) for recovery[a] Stratification by cohort Excluding COPDGene, SHS	Hazard ratio (95% CI) for recovery[a] Stratification by cohort Excluding COPDGene, PrePF, SARP, SPIROMICS
Age (ref: <50 years)	50 – 64 years	1.04 (0.94, 1.15)	1.02 (0.92, 1.12)
	65 – 79 years	1.02 (0.90, 1.15)	1.03 (0.91, 1.17)
	80+ years	1.02 (0.84, 1.24)	1.01 (0.83, 1.22)
Sex (ref: men)	Women	0.84 (0.78, 0.91)	0.84 (0.78, 0.90)
Educational attainment (ref: College)	< High School	1.04 (0.93, 1.16)	1.04 (0.93, 1.16)
	High School	1.02 (0.93, 1.13)	1.02 (0.93, 1.12)
	Some College	1.01 (0.91, 1.13)	1.03 (0.93, 1.15)
Smoking Status (ref: Never)	Former	0.97 (0.90, 1.06)	0.95 (0.88, 1.04)
	Current	0.94 (0.84, 1.07)	0.92 (0.82, 1.04)
BMI (ref: <25 kg/m^2)	25- <30 kg/m^2	1.01 (0.91, 1.11)	1.01 (0.91, 1.11)
	30+ kg/m^2	0.90 (0.81, 1.00)	0.91 (0.82, 1.00)
Diabetes (ref: absent)	Present	0.94 (0.85, 1.05)	0.95 (0.87, 1.04)
Hypertension (ref: Absent)	Present	0.95 (0.87, 1.04)	0.95 (0.87, 1.04)
Cardiovascular disease (ref: absent)	Present	0.85 (0.71, 1.01)	0.83 (0.70, 1.00)
eGFR < 45 ml/min/1.73m^2 (ref: eGFR ≥45 ml/min/1.73m^2)	Present	0.91 (0.73, 1.12)	0.90 (0.72, 1.11)
Asthma (ref: absent)	Present	0.99 (0.89, 1.10)	1.00 (0.90, 1.12)
COPD (ref: absent)	Present	0.91 (0.76, 1.09)	0.87 (0.72, 1.11)
Elevated depressive symptoms (ref: absent)	Present	0.92 (0.82, 1.03)	0.91 (0.82, 1.02)
Vaccination prior to infection (ref: no)	Yes	1.27 (1.08, 1.48)	1.24 (1.06, 1.45)
Infection wave (ref: 1st, WT, Spring 2020)	Second (WT, Summer/Fall 2020)	1.01 (0.89, 1.14)	1.00 (0.88, 1.13)
	Third (Alpha, Winter 2020-21)	0.94 (0.85, 1.04)	0.92 (0.83, 1.02)
	Fourth (Spring 2021)	0.90 (0.75, 1.07)	0.88 (0.74, 1.05)
	Fifth (Delta, Summer 2021)	0.95 (0.81, 1.12)	0.93 (0.79, 1.10)
	Sixth (Omicron, Winter 2021-present)	1.21 (1.01, 1.45)	1.19 (1.00, 1.42)

[a]Cox proportional hazards models were performed to assess associations of time-to-recovery with the correlates of interest. Models treated cohort as a stratum term, allowing each cohort to have its own baseline hazard function. Estimates were generated from models adjusted for all of the covariates listed in the table. Hazards ratios (HRs) greater than one indicate faster recovery, whereas HRs less than one indicate slower recovery.

© 2024 Oelsner EC et al. *JAMA Network Open.*

eFigure 1. CONSORT Diagram of Participants Included in Analyses. Target population includes cohort participants believed to be alive and not lost-to-follow-up on March 1, 2020. *Selected participants did complete a C4R data element but not consent to data sharing on the C4R Analysis Commons by the time of manuscript preparation; these participants are included in N=3,362.

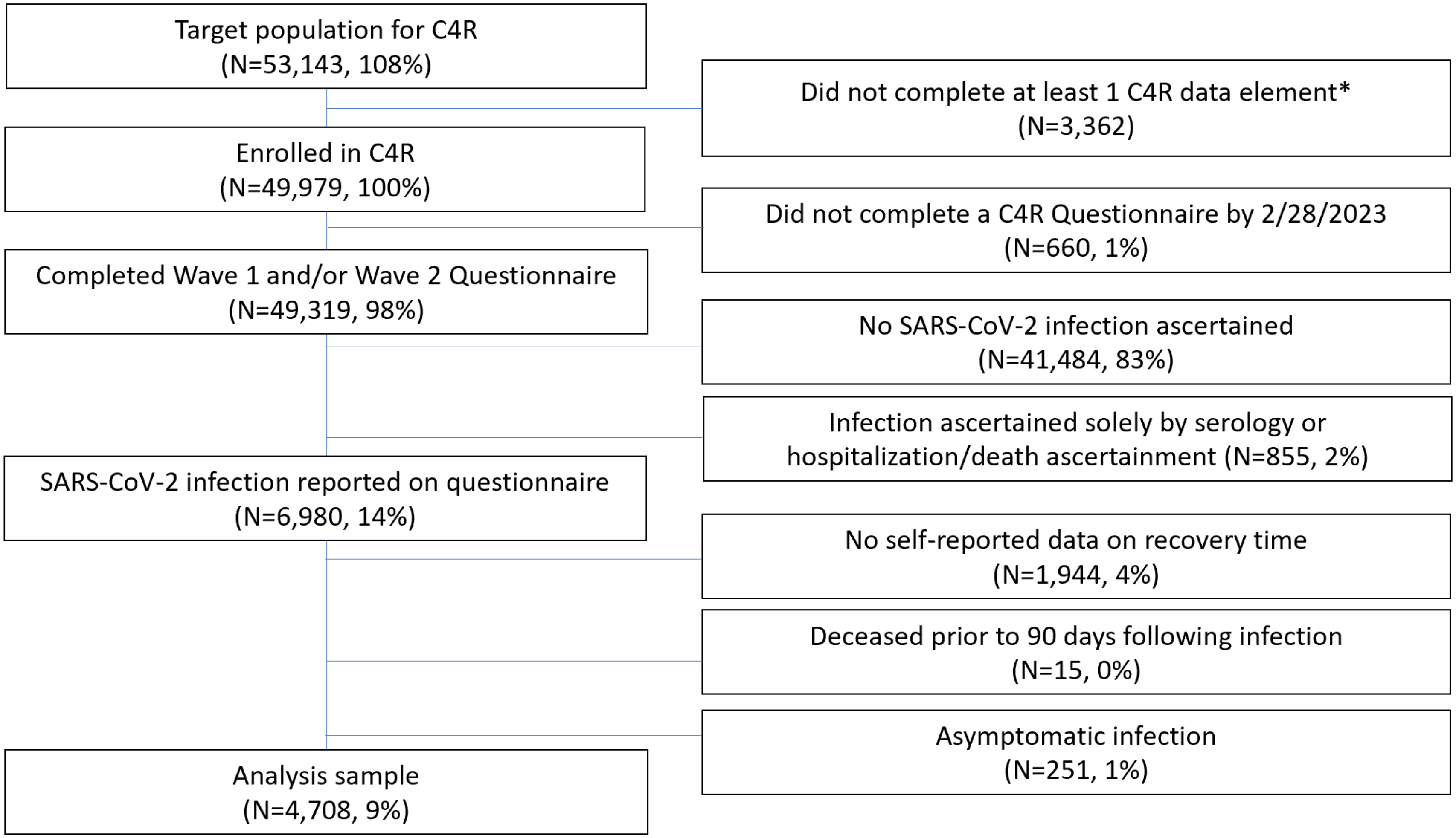

© 2024 Oelsner EC et al. *JAMA Network Open.*

eFigure 2. Correlates of Recovery by 90 Days After SARS-CoV-2 Infection, After Multivariable Adjustment, Stratified by Vaccination Status at Time of Infection. Effect estimates for unvaccinated cases indicated by empty dots, and effect estimates for vaccinated cases indicated by black dots. Correlates that were significantly associated in the main model are highlighted in orange. There were no statistically significant interaction terms for vaccination status. Null effect (HR = 1.0) indicated by red line. CKD = eGFR < 45 ml/min/1.73m[2]

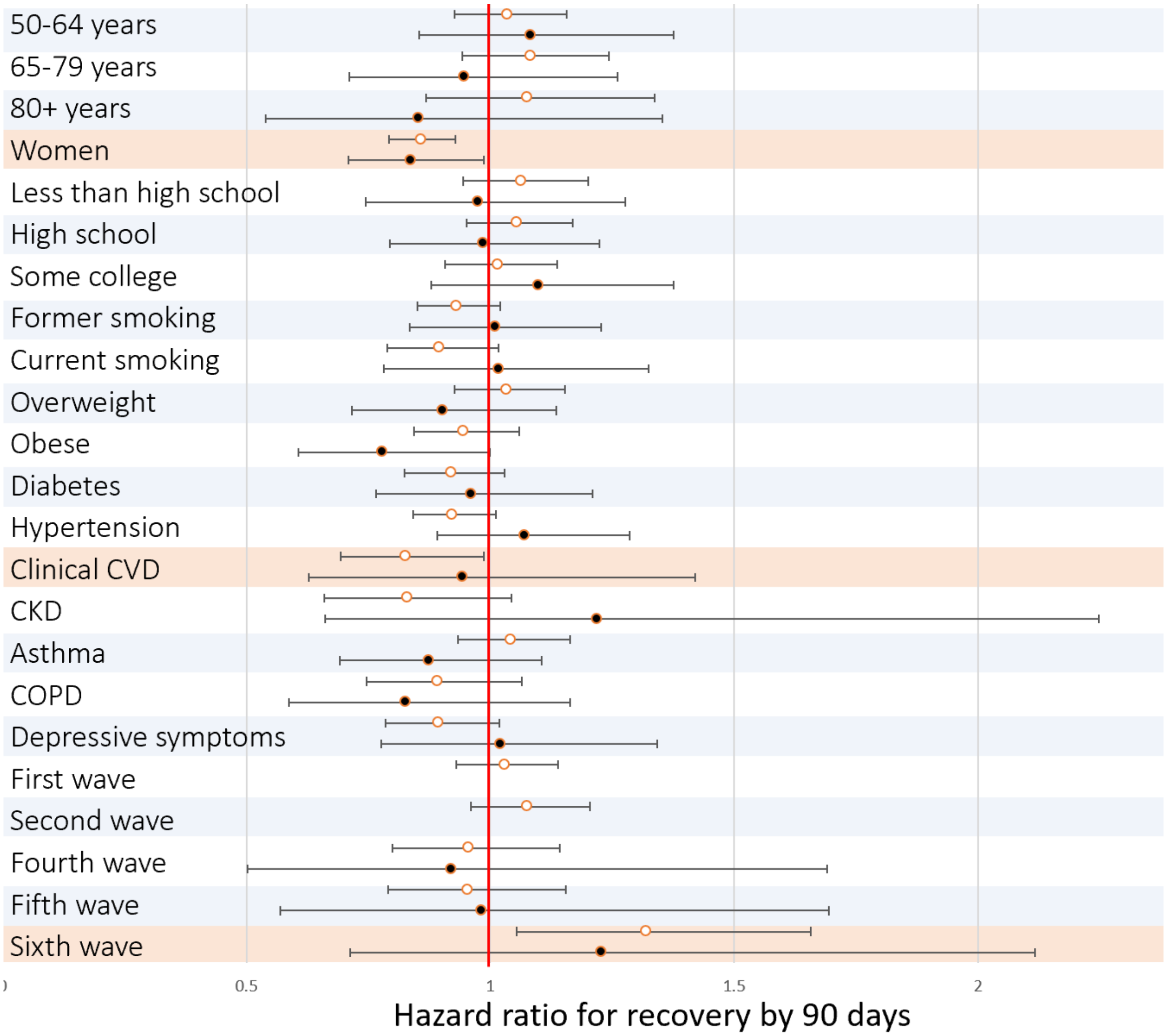

© 2024 Oelsner EC et al. *JAMA Network Open.*

eFigure 3. Restricted Mean Recovery Time in Days Following SARS-CoV-2 Infection, by Cohort.

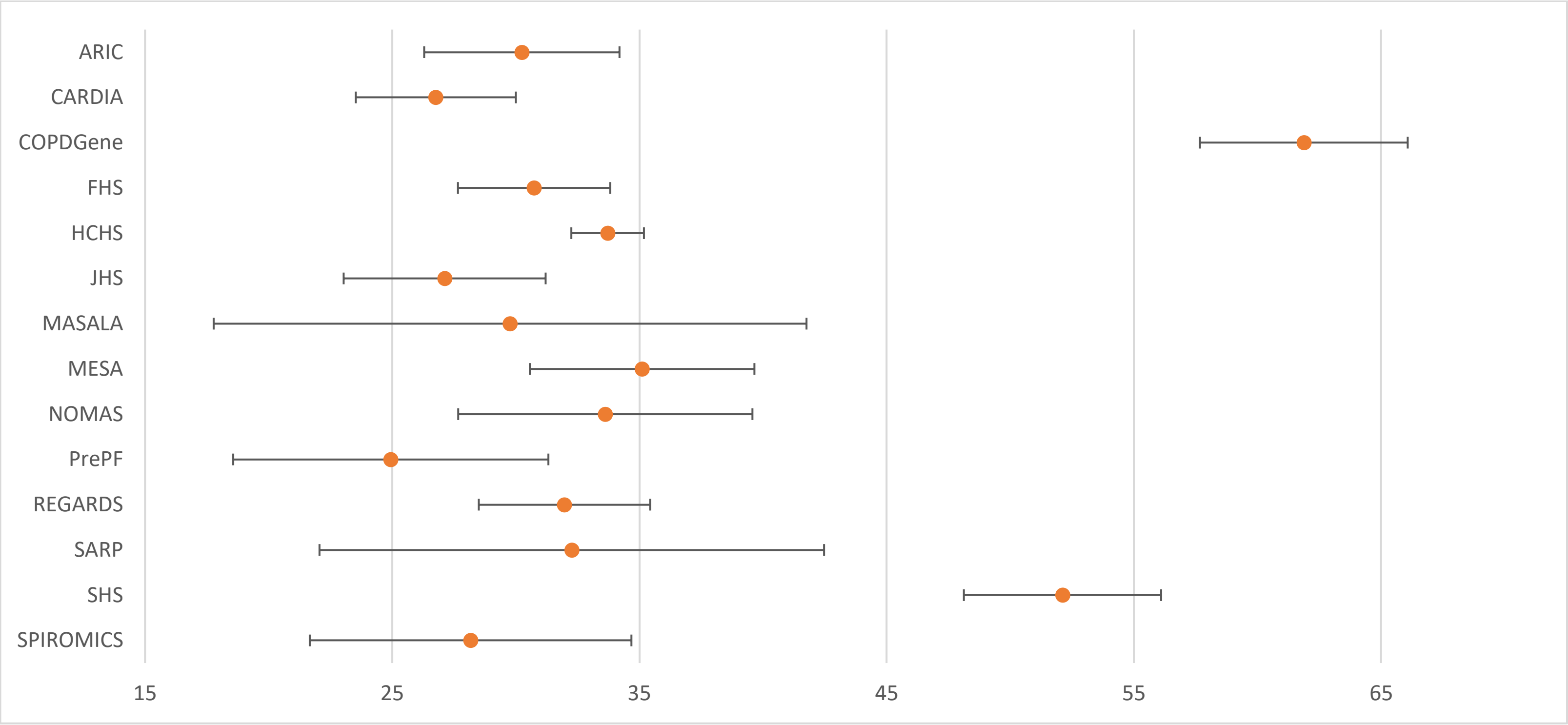

© 2024 Oelsner EC et al. *JAMA Network Open.*

eReferences

1. The Atherosclerosis Risk in Communities (ARIC) Study: design and objectives. The ARIC investigators. *Am J Epidemiol*. Apr 1989;129(4):687-702.
2. Friedman GD, Cutter GR, Donahue RP, et al. CARDIA: study design, recruitment, and some characteristics of the examined subjects. *J Clin Epidemiol*. 1988;41(11):1105-16. doi:10.1016/0895-4356(88)90080-7
3. Regan EA, Hokanson JE, Murphy JR, et al. Genetic epidemiology of COPD (COPDGene) study design. *COPD*. Feb 2010;7(1):32-43. doi:10.3109/15412550903499522
4. Tsao CW, Vasan RS. Cohort Profile: The Framingham Heart Study (FHS): overview of milestones in cardiovascular epidemiology. *Int J Epidemiol*. Dec 2015;44(6):1800-13. doi:10.1093/ije/dyv337
5. Daviglus ML, Talavera GA, Aviles-Santa ML, et al. Prevalence of major cardiovascular risk factors and cardiovascular diseases among Hispanic/Latino individuals of diverse backgrounds in the United States. *JAMA*. Nov 7 2012;308(17):1775-84. doi:10.1001/jama.2012.14517
6. Lavange LM, Kalsbeek WD, Sorlie PD, et al. Sample design and cohort selection in the Hispanic Community Health Study/Study of Latinos. *Ann Epidemiol*. Aug 2010;20(8):642-9. doi:10.1016/j.annepidem.2010.05.006
7. Sorlie PD, Aviles-Santa LM, Wassertheil-Smoller S, et al. Design and implementation of the Hispanic Community Health Study/Study of Latinos. *Ann Epidemiol*. Aug 2010;20(8):629-41. doi:10.1016/j.annepidem.2010.03.015
8. Carpenter MA, Crow R, Steffes M, et al. Laboratory, reading center, and coordinating center data management methods in the Jackson Heart Study. *Am J Med Sci*. Sep 2004;328(3):131-44. doi:10.1097/00000441-200409000-00001
9. Keku E, Rosamond W, Taylor HA, Jr., et al. Cardiovascular disease event classification in the Jackson Heart Study: methods and procedures. *Ethn Dis*. Autumn 2005;15(4 Suppl 6):S6-62-70.
10. Taylor HA, Jr., Wilson JG, Jones DW, et al. Toward resolution of cardiovascular health disparities in African Americans: design and methods of the Jackson Heart Study. *Ethn Dis*. Autumn 2005;15(4 Suppl 6):S6-4-17.
11. Kanaya AM, Chang A, Schembri M, et al. Recruitment and retention of US South Asians for an epidemiologic cohort: Experience from the MASALA study. *J Clin Transl Sci*. Jun 2019;3(2-3):97-104. doi:10.1017/cts.2019.371
12. Kanaya AM, Kandula N, Herrington D, et al. Mediators of Atherosclerosis in South Asians Living in America (MASALA) study: objectives, methods, and cohort description. *Clin Cardiol*. Dec 2013;36(12):713-720. doi:10.1002/clc.22219
13. Bild DE, Bluemke DA, Burke GL, et al. Multi-Ethnic Study of Atherosclerosis: objectives and design. *Am J Epidemiol*. Nov 1 2002;156(9):871-81. doi:10.1093/aje/kwf113
14. Sacco RL, Boden-Albala B, Gan R, et al. Stroke incidence among white, black, and Hispanic residents of an urban community: the Northern Manhattan Stroke Study. *Am J Epidemiol*. Feb 1 1998;147(3):259-68. doi:10.1093/oxfordjournals.aje.a009445
15. Howard VJ, Cushman M, Pulley L, et al. The reasons for geographic and racial differences in stroke study: objectives and design. *Neuroepidemiology*. 2005;25(3):135-43. doi:10.1159/000086678
16. Teague WG, Phillips BR, Fahy JV, et al. Baseline Features of the Severe Asthma Research Program (SARP III) Cohort: Differences with Age. *J Allergy Clin Immunol Pract*. Mar - Apr 2018;6(2):545-554 e4. doi:10.1016/j.jaip.2017.05.032
17. Couper D, LaVange LM, Han M, et al. Design of the Subpopulations and Intermediate Outcomes in COPD Study (SPIROMICS). *Thorax*. May 2014;69(5):491-4. doi:10.1136/thoraxjnl-2013-203897

© 2024 Oelsner EC et al. *JAMA Network Open.*

18. Lee ET, Welty TK, Fabsitz R, et al. The Strong Heart Study. A study of cardiovascular disease in American Indians: design and methods. *Am J Epidemiol*. Dec 1990;132(6):1141-55. doi:10.1093/oxfordjournals.aje.a115757
19. North KE, Howard BV, Welty TK, et al. Genetic and environmental contributions to cardiovascular disease risk in American Indians: the strong heart family study. *Am J Epidemiol*. Feb 15 2003;157(4):303-14. doi:10.1093/aje/kwf208
20. Oelsner EC, Krishnaswamy A, Balte PP, et al. Collaborative Cohort of Cohorts for COVID-19 Research (C4R) Study: Study Design. *Am J Epidemiol*. Jun 27 2022;191(7):1153-1173. doi:10.1093/aje/kwac032
21. Delanaye P, Glassock RJ, Pottel H, Rule AD. An Age-Calibrated Definition of Chronic Kidney Disease: Rationale and Benefits. *Clin Biochem Rev*. Feb 2016;37(1):17-26.
22. White IR, Royston P. Imputing missing covariate values for the Cox model. *Stat Med*. Jul 10 2009;28(15):1982-98. doi:10.1002/sim.3618
23. van Buuren S, Groothuis-Oudshoorn K. mice: Multivariate Imputation by Chained Equations in R. *Journal of Statistical Software*. 2011;45(3):1-67. doi:doi:10.18637/jss.v045.i03.

© 2024 Oelsner EC et al. *JAMA Network Open.*
