# Supplementary material for: Epidemiologic Features of Recovery From SARS-CoV-2 Infection
Source: JAMA Netw Open. 2024 Jun 17;7(6):e2417440. doi: 10.1001/jamanetworkopen.2024.17440 (PMC11184459; doi:10.1001/jamanetworkopen.2024.17440)
Supplement: Supplement 2. — Data Sharing Statement [file jamanetwopen-e2417440-s002.pdf]

Data Sharing Statement

Oelsner. Epidemiologic Features of Recovery From SARS-CoV-2 Infection in a US Population–Based Cohort. *JAMA Netw Open*. Published June 17, 2024.
doi:10.1001/jamanetworkopen.2024.17440

Data
Data available: Yes
Data types: Deidentified participant data, Data dictionary
How to access data: Data requests should be sent to c4r@cumc.columbia.edu
When available: With publication

Supporting Documents
Document types: None

Additional Information
Who can access the data: Data will be made available to researchers whose proposed use of the data has been approved.
Types of analyses: For a specified and approved purpose.
Mechanisms of data availability: After approval of a proposal.
